# Supplementary material for: Activation of a Sweet Taste Receptor by Oleanane-Type Glycosides from Wisteria sinensis
Source: Molecules. 2022 Nov 15;27(22):7866. doi: 10.3390/molecules27227866 (PMC9699193; doi:10.3390/molecules27227866)
Supplement: Supplementary file 1 [file molecules-27-07866-s001.zip › molecules-2009906-supplementary.pdf]

Supplementary data

Activation of a sweet taste receptor by oleanane-type glycosides from *Wisteria sinensis*

Samir Hobloss[1], Antoine Bruguière[1], David Pertuit[1], Tomofumi Miyamoto[2], Chiaki Tanaka[2], Christine Belloir[1], Marie-Aleth Lacaille-Dubois[1], Loïc Briand[1], Anne-Claire Mitaine-Offer[1,*].

[1] Centre des Sciences du Goût et de l'Alimentation, CNRS, INRAE, Institut Agro, Université de Bourgogne Franche-Comté, 21000 Dijon, France

[2] Graduate School of Pharmaceutical Sciences, Kyushu University, Fukuoka, 812-8582, Japan

* Correspondence: anne-claire.offer@u-bourgogne.fr

Content

Figure S1 - ^{1}H spectrum of compound 1 3
Figure S2 - ^{1}H spectrum of compound 1 zoomed-in (a) 4
Figure S3 - ^{1}H spectrum of compound 1 zoomed-in (b) 5
Figure S4 - ^{13}C spectrum of compound 1 6
Figure S5 - ^{13}C spectrum of compound 1 zoomed-in (a) 7
Figure S6 - ^{13}C spectrum of compound 1 zoomed-in (b) 8
Figure S7 - ^{13}C spectrum of compound 1 zoomed-in (c) 9
Figure S8 - HSQC spectrum of compound 1 10
Figure S9 - HSQC spectrum of compound 1 zoomed-in (a) 11
Figure S10 - HSQC spectrum of compound 1 zoomed-in (b) 12
Figure S11 - HSQC spectrum of compound 1 zoomed-in (c) 13
Figure S12 - HMBC spectrum of compound-in 1 14
Figure S13 - HMBC spectrum of compound 1 zoomed-in 15
Figure S14 - COSY spectrum of compound 1 16
Figure S15 - COSY spectrum of compound 1 zoomed-in (a) 17
Figure S16 - COSY spectrum of compound 1 zoomed-in (b) 18
Figure S17 - ROESY spectrum of compound 1 19
Figure S18 - ROESY spectrum of compound 1 zoomed-in (a) 20
Figure S19 - ROESY spectrum of compound 1 zoomed-in (b) 21
Figure S20 - ^{1}H spectrum of compound 2 22
Figure S21 - ^{1}H spectrum of compound 2 zoomed-in (a) 23
Figure S22 - ^{1}H spectrum of compound 2 zoomed-in (b) 24
Figure S23 - ^{13}C spectrum of compound 2 25
Figure S24 - ^{13}C spectrum of compound 2 zoomed-in (a) 26
Figure S25 - ^{13}C spectrum of compound 2 zoomed-in (b) 27
Figure S26 - ^{13}C spectrum of compound 2 zoomed-in (c) 28
Figure S27 - HSQC spectrum of compound 2 29
Figure S28 - HSQC spectrum of compound 2 zoomed-in (a) 30
Figure S29- HSQC spectrum of compound 2 zoomed-in (b) 31
Figure S30 - HSQC spectrum of compound 2 zoomed-in (c) 32
Figure S31 - HMBC spectrum of compound 2 33
Figure S32 - HMBC spectrum of compound 2 zoomed-in (a) 34
Figure S33 - HMBC spectrum of compound 2 zoomed-in (b) 35
Figure S34 - HMBC spectrum of compound 2 zoomed-in (c) 36
Figure S35 - COSY spectrum of compound 2 37
Figure S36 - COSY spectrum of compound 2 zoomed-in 38
Figure S37 - ROESY spectrum of compound 2 39
Figure S38 - ROESY spectrum of compound 2 zoomed-in 40

Compound 1

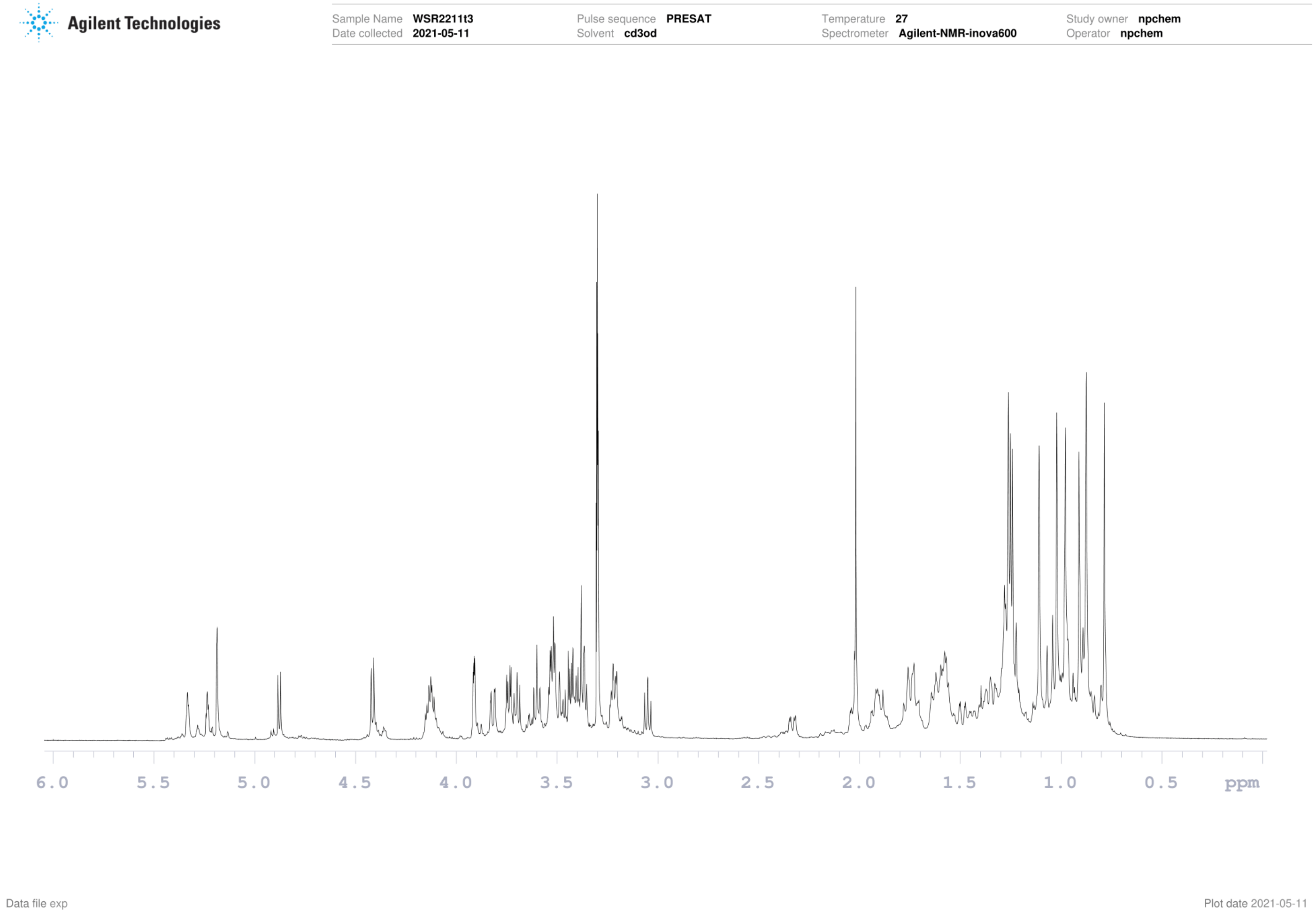

Figure S1 - ^{1}H spectrum of compound 1

Compound 1

Sample Name	WSR2211t3	Pulse sequence	PRESAT	Temperature	27	Study owner	npchem
Date collected	2021-05-11	Solvent	cd3od	Spectrometer	Agilent-NMR-inova600	Operator	npchem

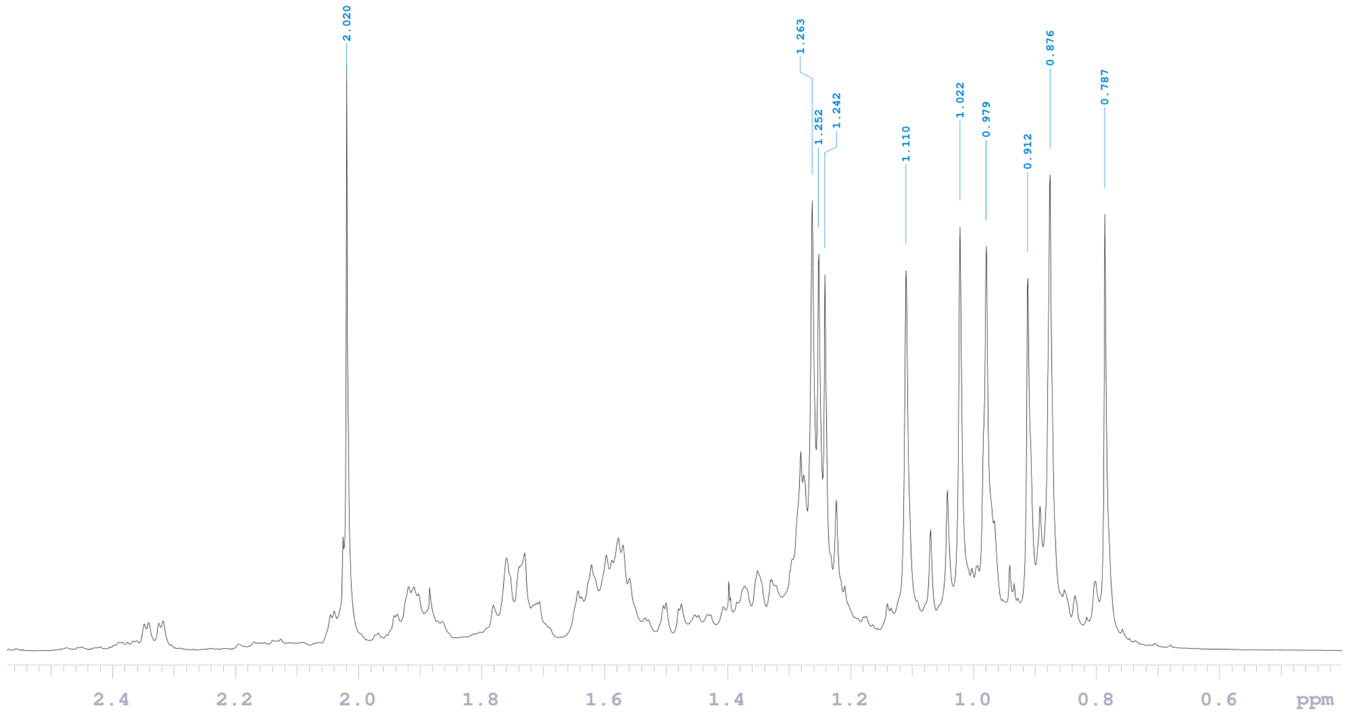

Data file exp

Plot date 2021-05-11

Figure S2 - ^{1}H spectrum of compound 1 zoomed-in (a)

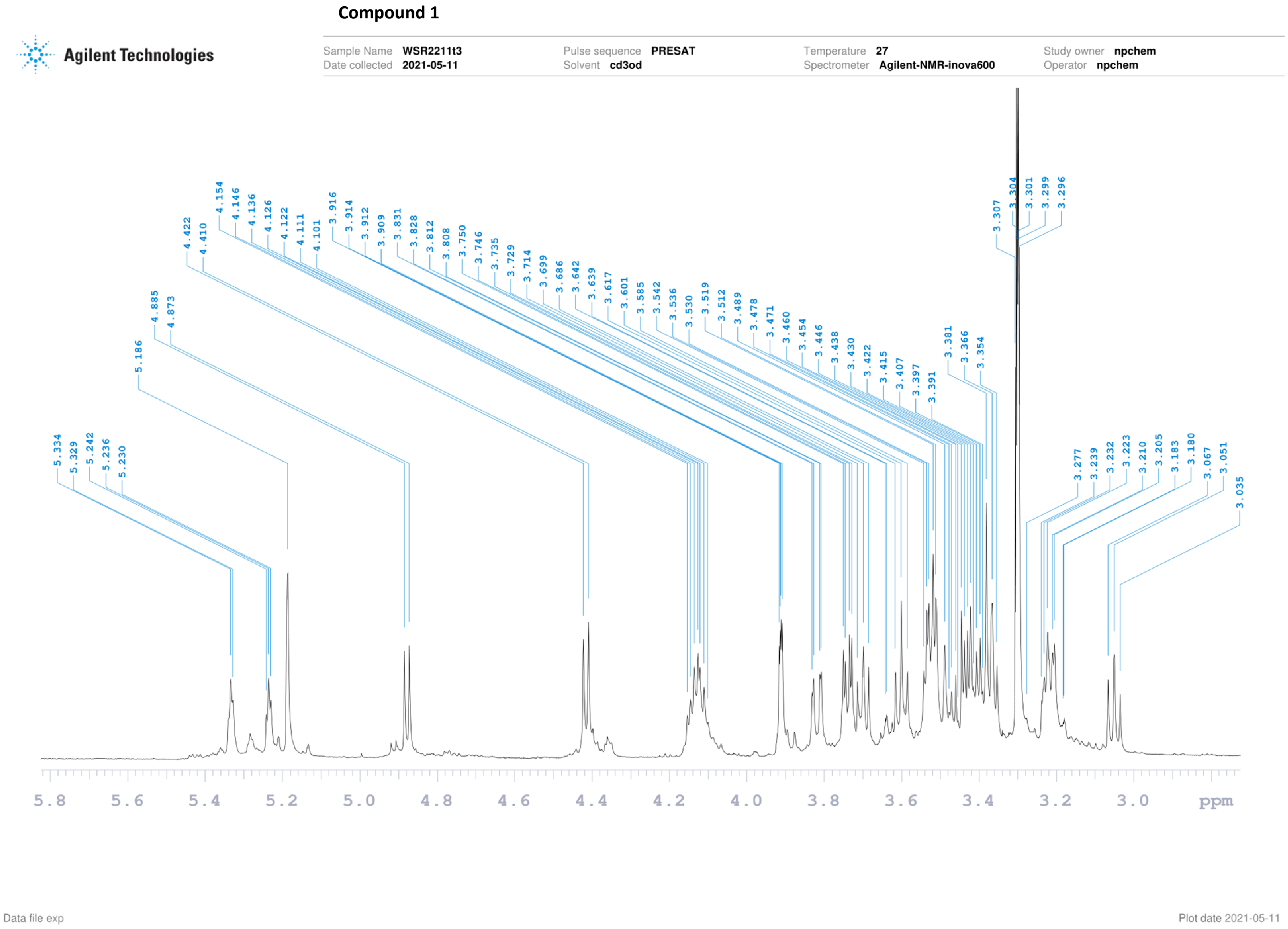

Figure S3 - ^{1}H spectrum of compound 1 zoomed-in (b)

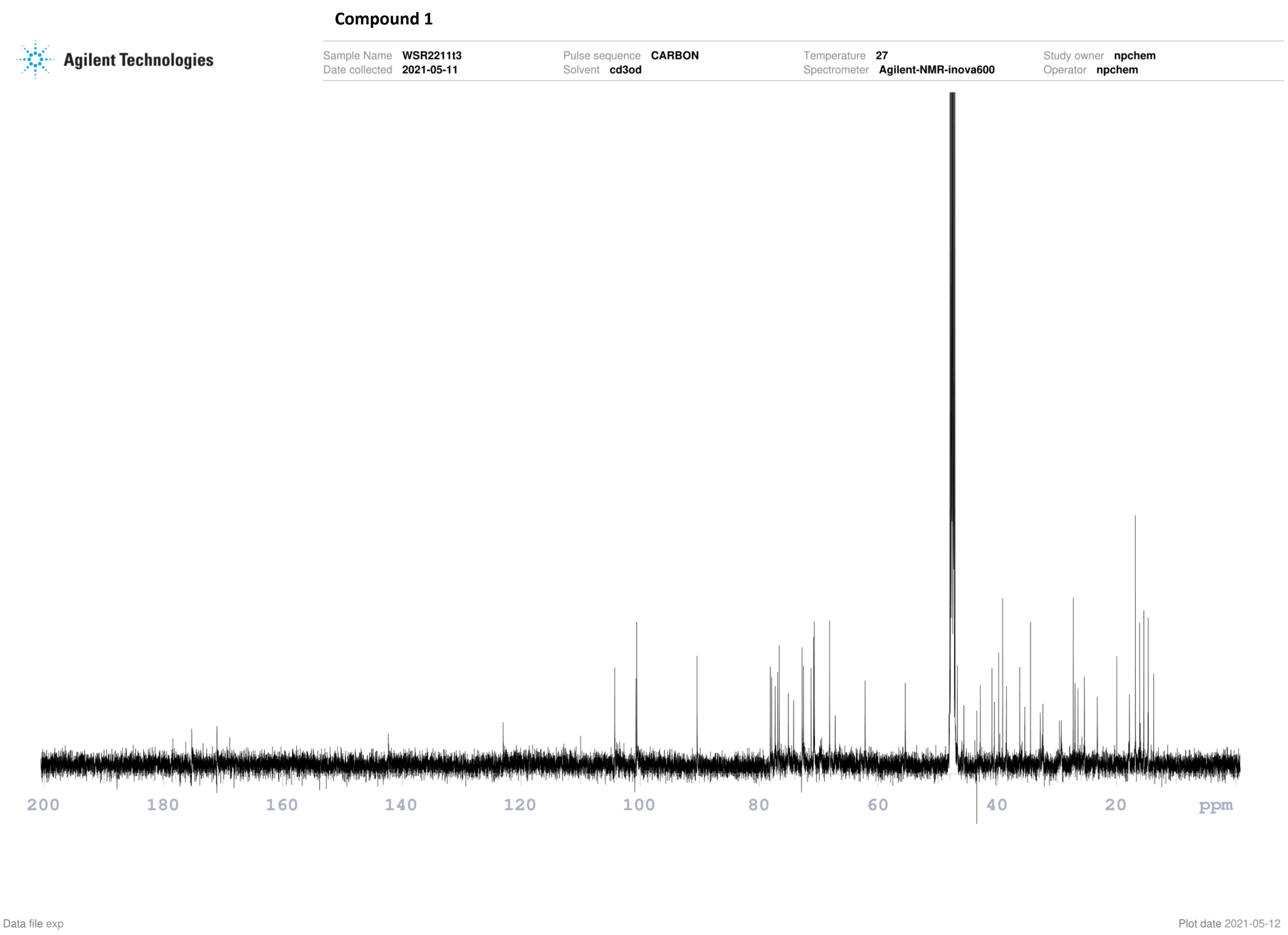

Figure S4 - ^{13}C spectrum of compound 1

Compound 1

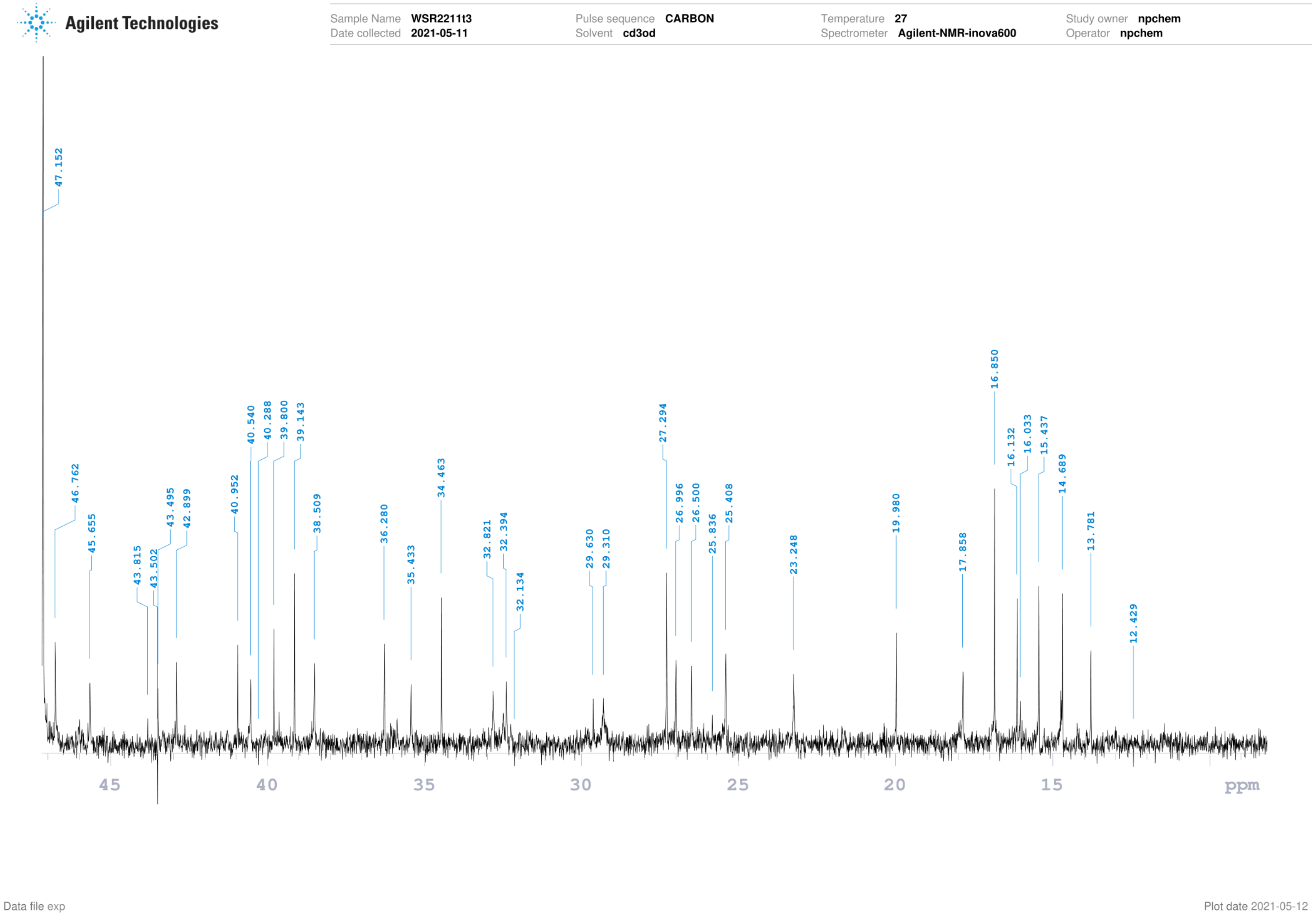

Figure S5 - ^{13}C spectrum of compound 1 zoomed-in (a)

Compound 1

Sample Name	WSR2211t3	Pulse sequence	CARBON	Temperature	27	Study owner	npchem
Date collected	2021-05-11	Solvent	cd3od	Spectrometer	Agilent-NMR-inova600	Operator	npchem

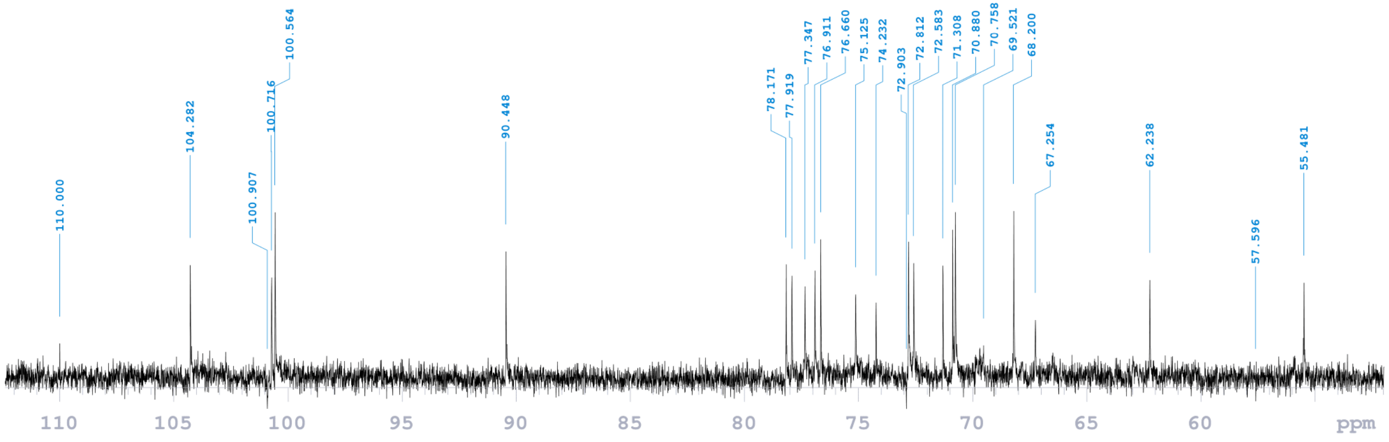

Data file exp

Plot date 2021-05-12

Figure S6 - ^{13}C spectrum of compound 1 zoomed-in (b)

Compound 1

Sample Name WSR2211t3 | Pulse sequence CARBON | Temperature 27 | Study owner npchem
Date collected 2021-05-11 | Solvent cd3od | Spectrometer Agilent-NMR-inova600 | Operator npchem

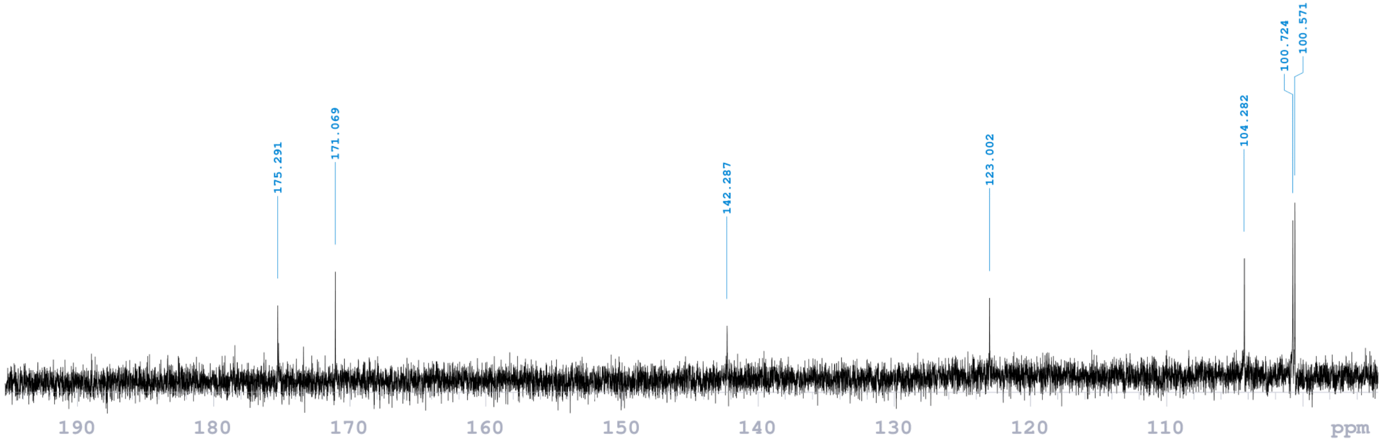

Data file exp

Plot date 2021-05-12

Figure S7 - ^{13}C spectrum of compound 1 zoomed-in (c)

Compound 1

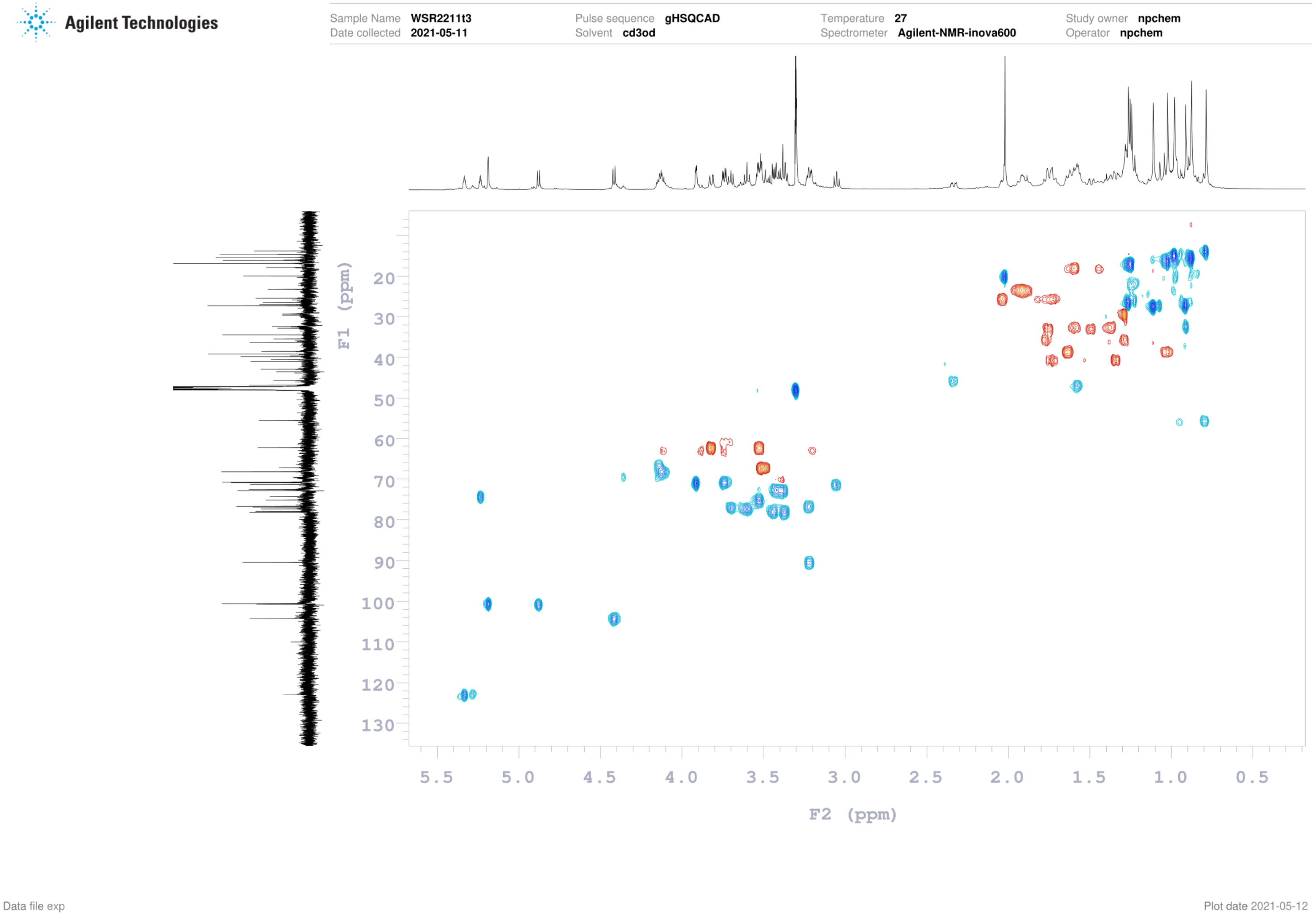

Figure S8 - HSQC spectrum of compound 1

Compound 1

Agilent Technologies

Sample Name WSR2211t3
Date collected 2021-05-11
Pulse sequence gHSQCAD
Solvent cd3od
Temperature 27
Spectrometer Agilent-NMR-inova600
Study owner npchem
Operator npchem

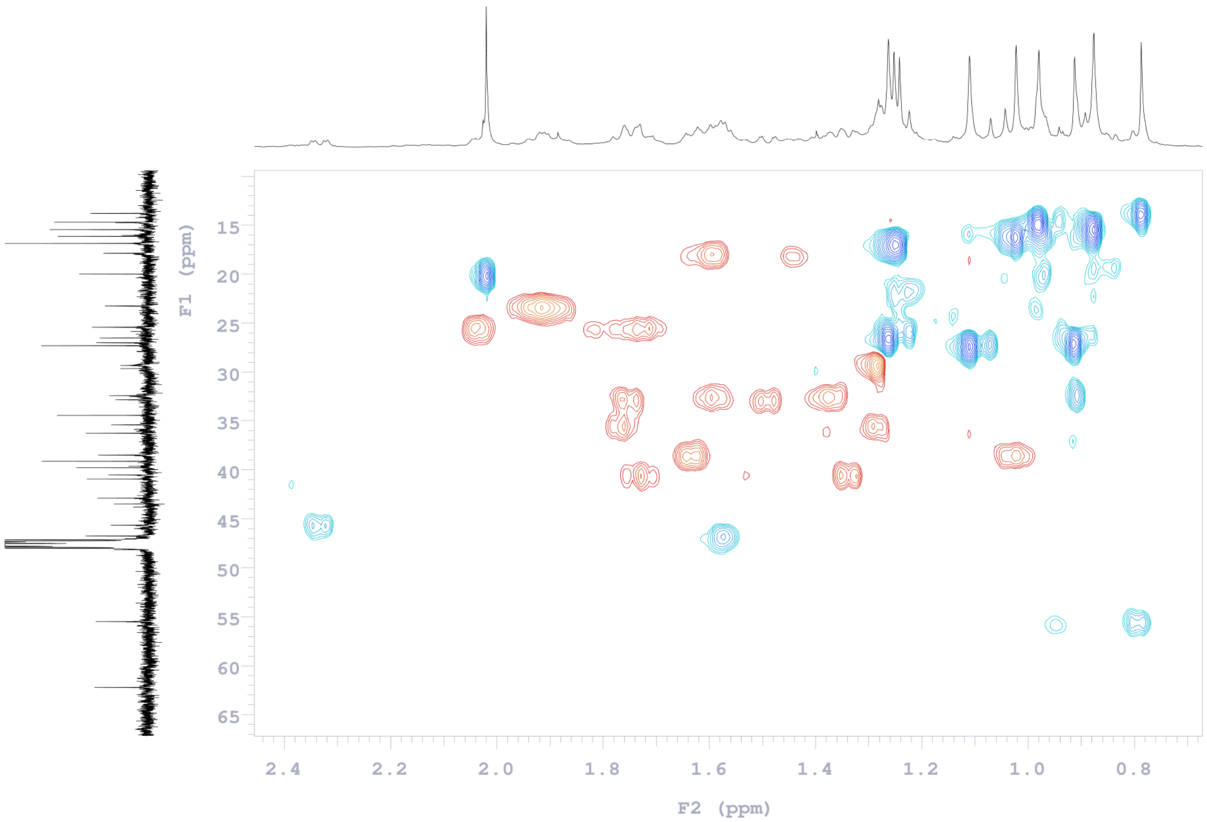

Data file exp

Plot date 2021-05-12

Figure S9 - HSQC spectrum of compound 1 zoomed-in (a)

Compound 1

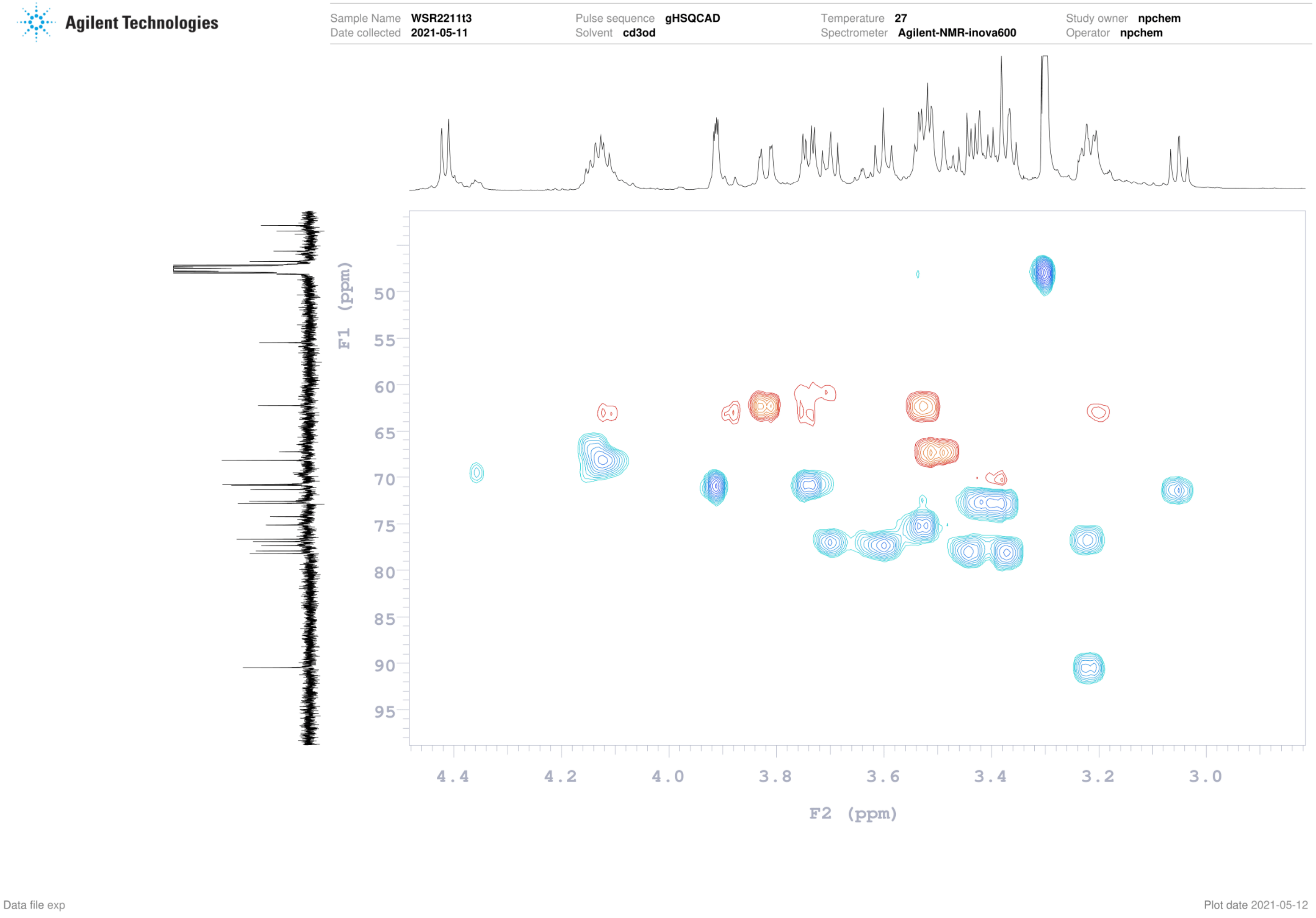

Figure S10 - HSQC spectrum of compound 1 zoomed-in (b)

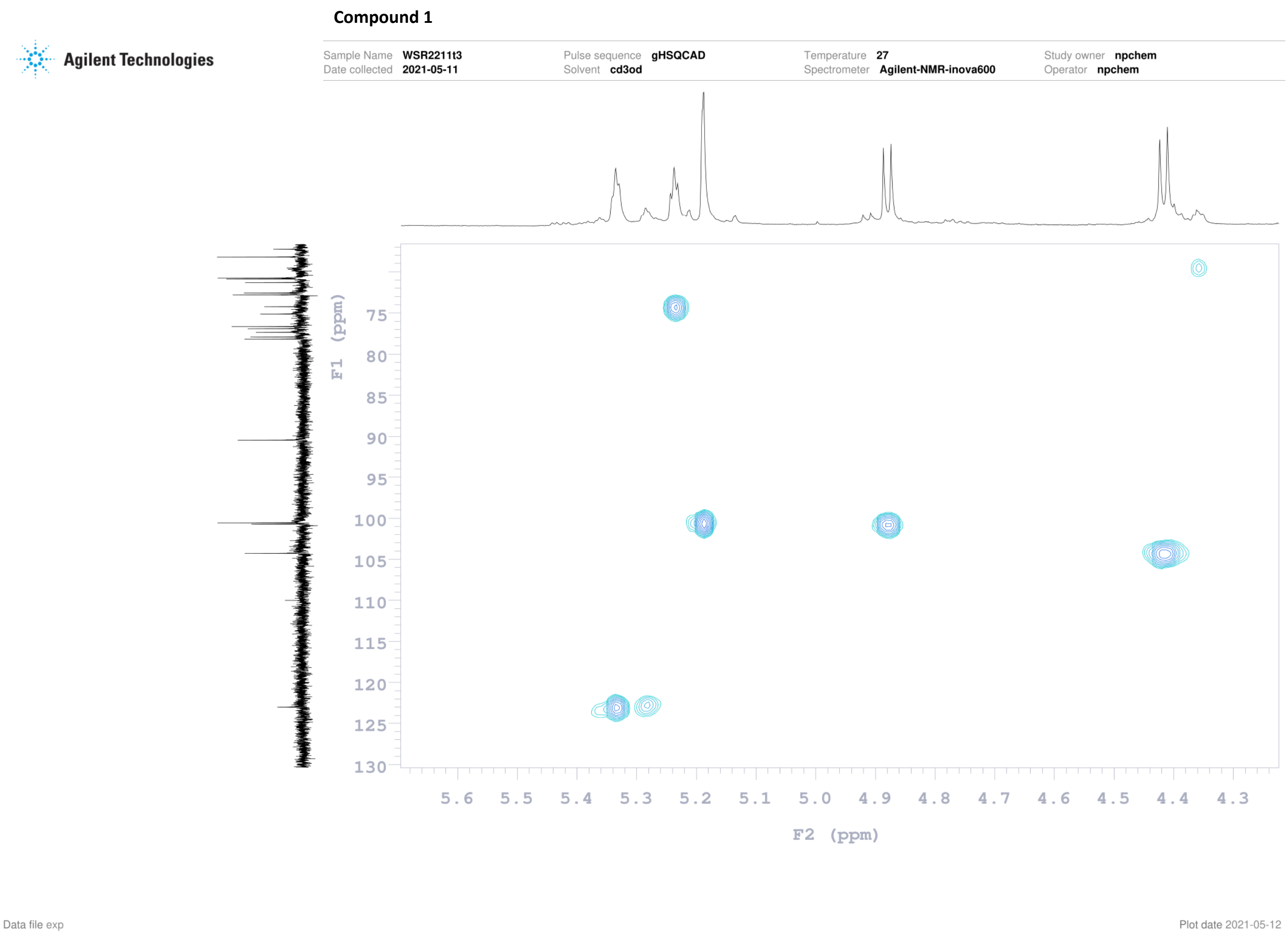

Figure S11 - HSQC spectrum of compound 1 zoomed-in (c)

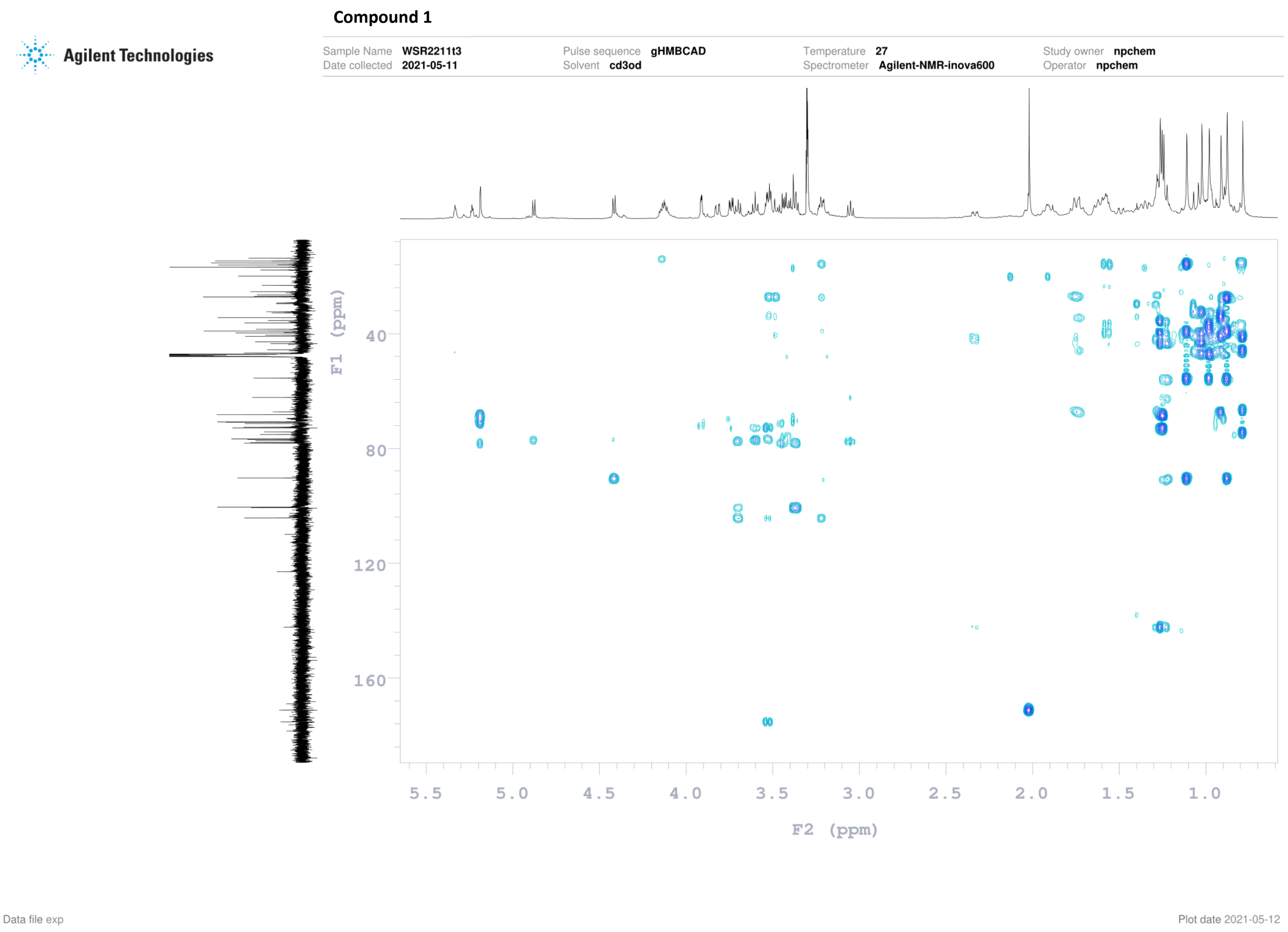

Figure S12 - HMBC spectrum of compound-in 1

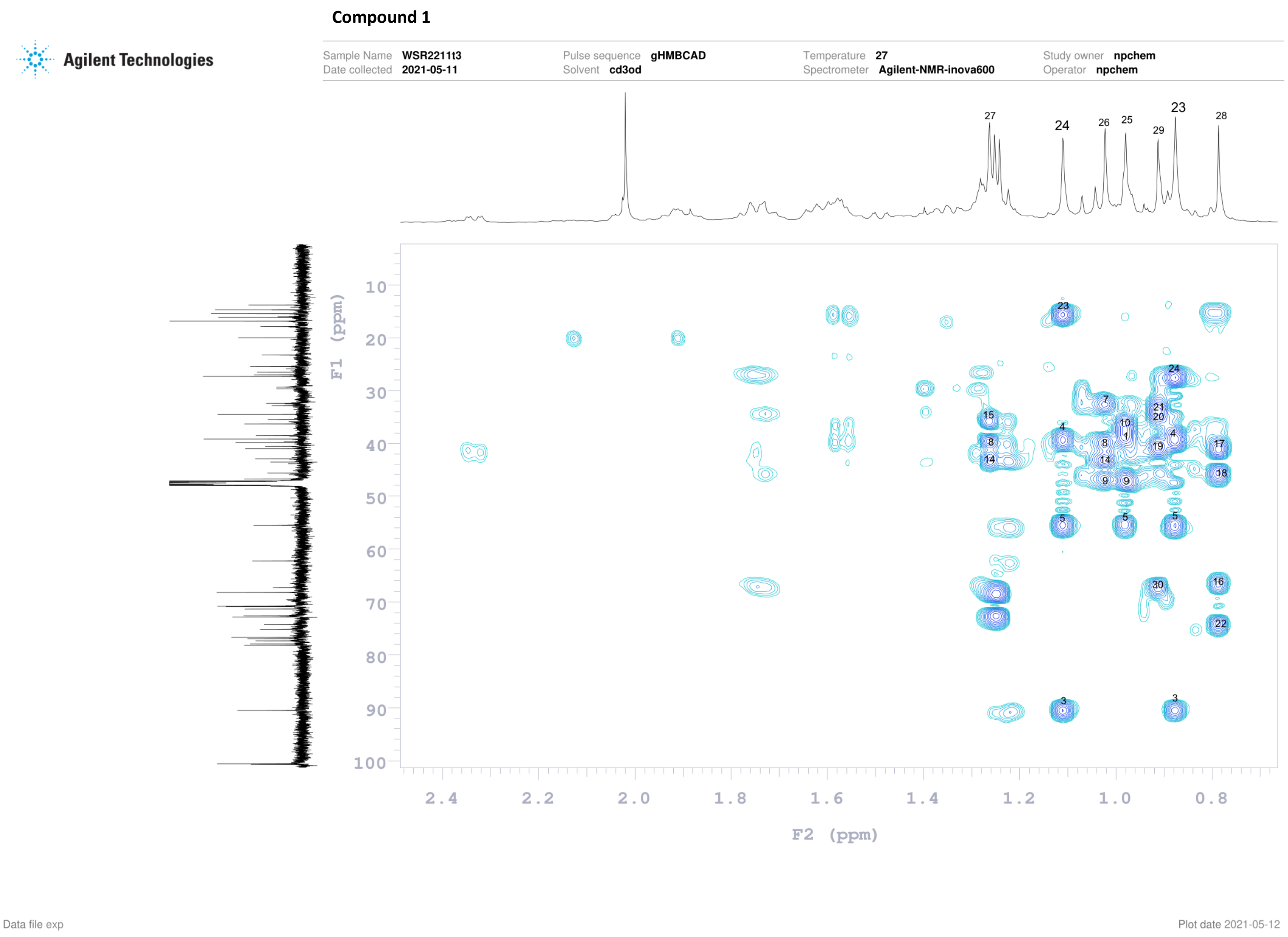

Figure S13 - HMBC spectrum of compound 1 zoomed-in

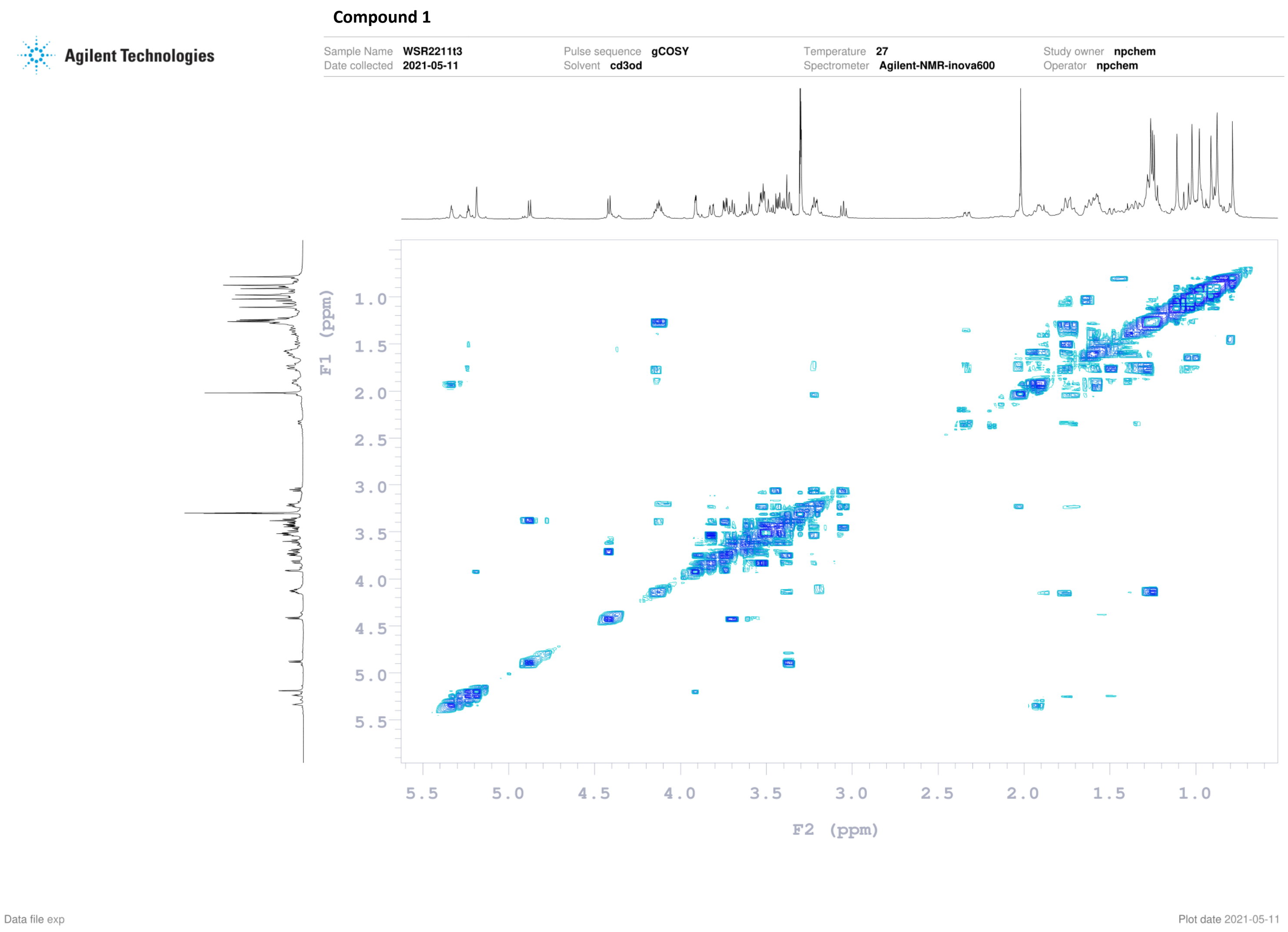

Figure S14 - COSY spectrum of compound 1

Compound 1

Sample Name	WSR2211t3	Pulse sequence	gCOSY	Temperature	27	Study owner	npchem
Date collected	2021-05-11	Solvent	cd3od	Spectrometer	Agilent-NMR-inova600	Operator	npchem

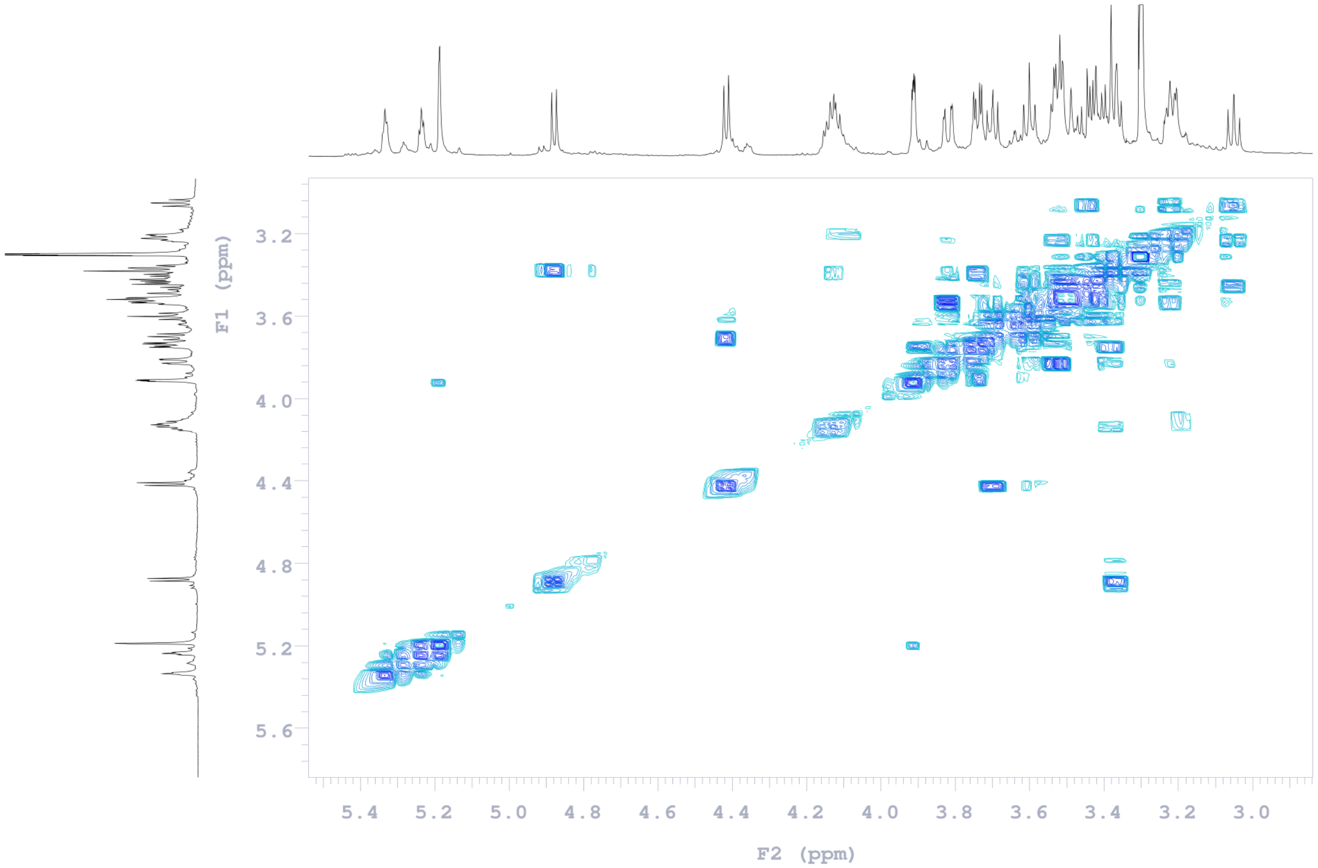

Data file exp

Plot date 2021-05-11

Figure S15 - COSY spectrum of compound 1 zoomed-in (a)

Compound 1

Sample Name	WSR2211t3	Pulse sequence	gCOSY	Temperature	27	Study owner	npchem
Date collected	2021-05-11	Solvent	cd3od	Spectrometer	Agilent-NMR-inova600	Operator	npchem

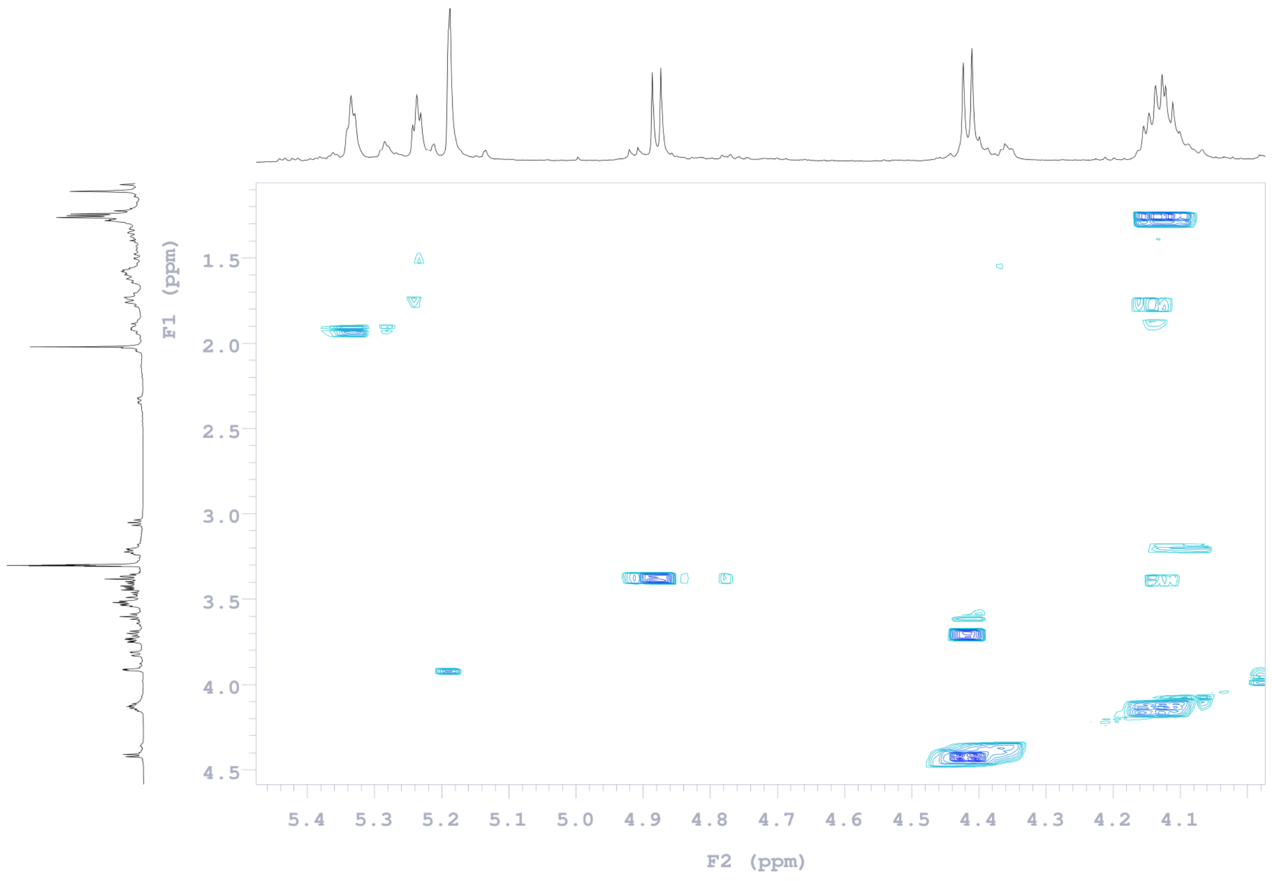

Data file exp

Plot date 2021-05-11

Figure S16 - COSY spectrum of compound 1 zoomed-in (b)

Compound 1

Agilent Technologies

Sample Name	WSR2211t3r	Pulse sequence	ROESYAD	Temperature	27	Study owner	npchem
Date collected	2021-05-18	Solvent	cd3od	Spectrometer	Agilent-NMR-inova600	Operator	npchem

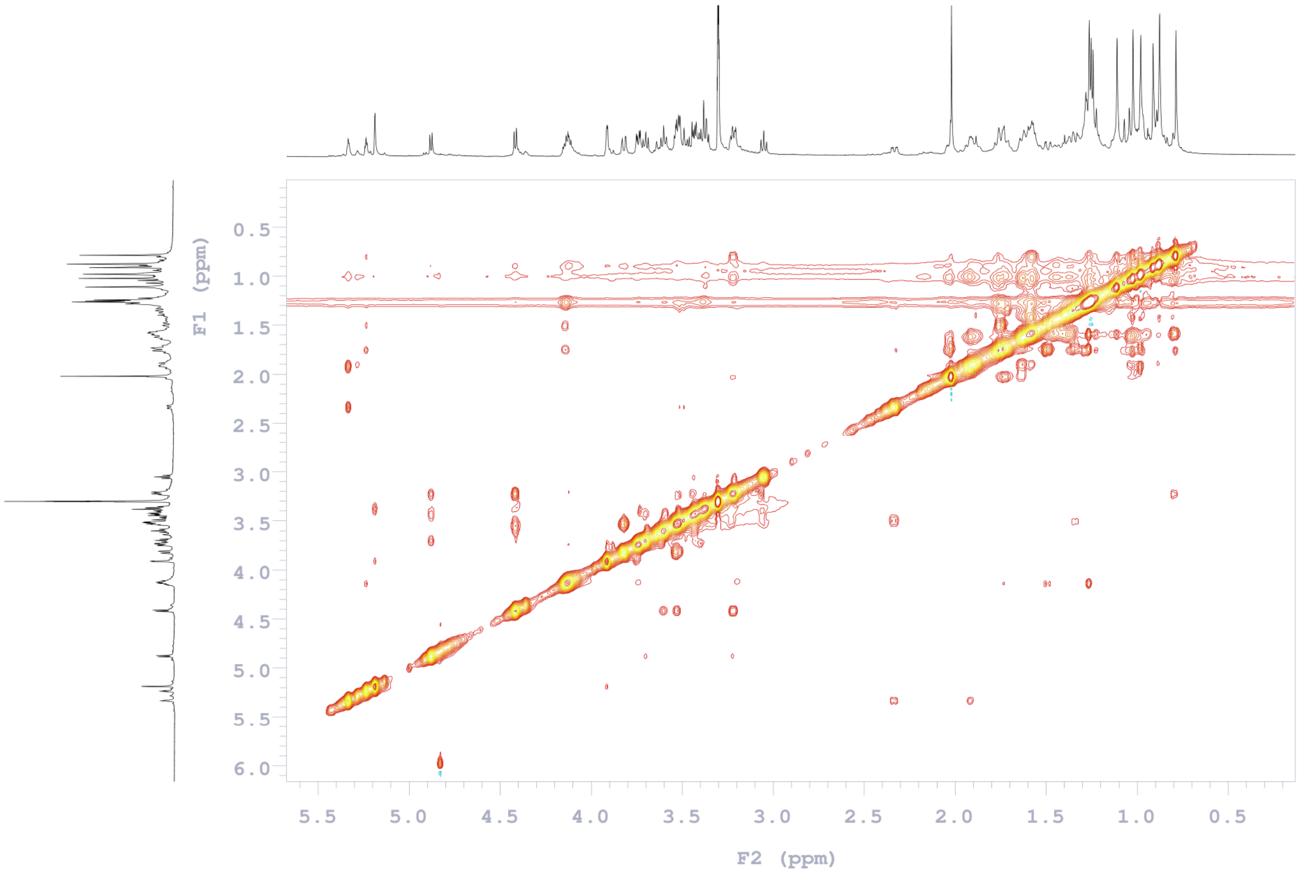

Data file exp

Plot date 2021-05-18

Figure S17 - ROESY spectrum of compound 1

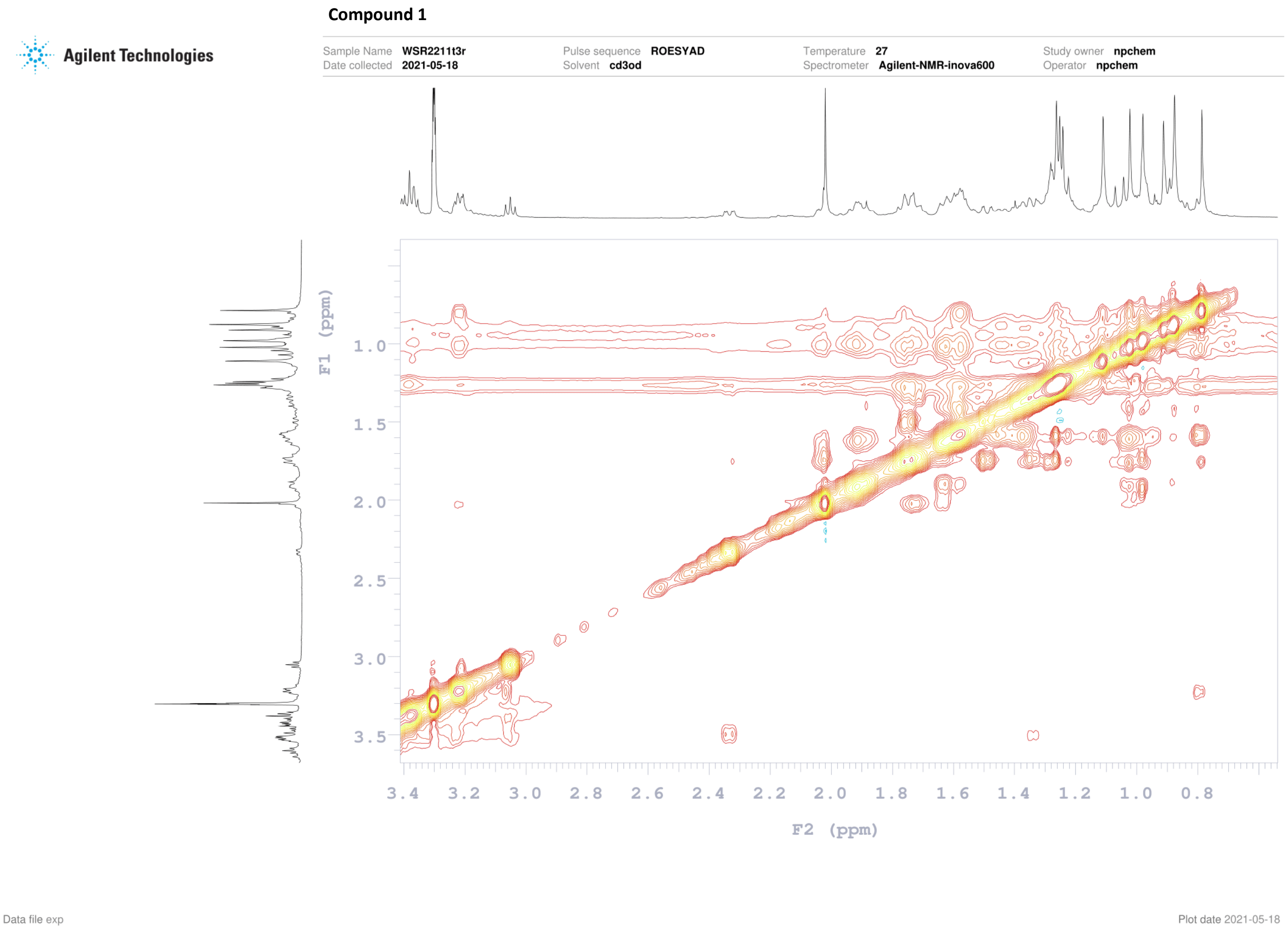

Figure S18 - ROESY spectrum of compound 1 zoomed-in (a)

Compound 1

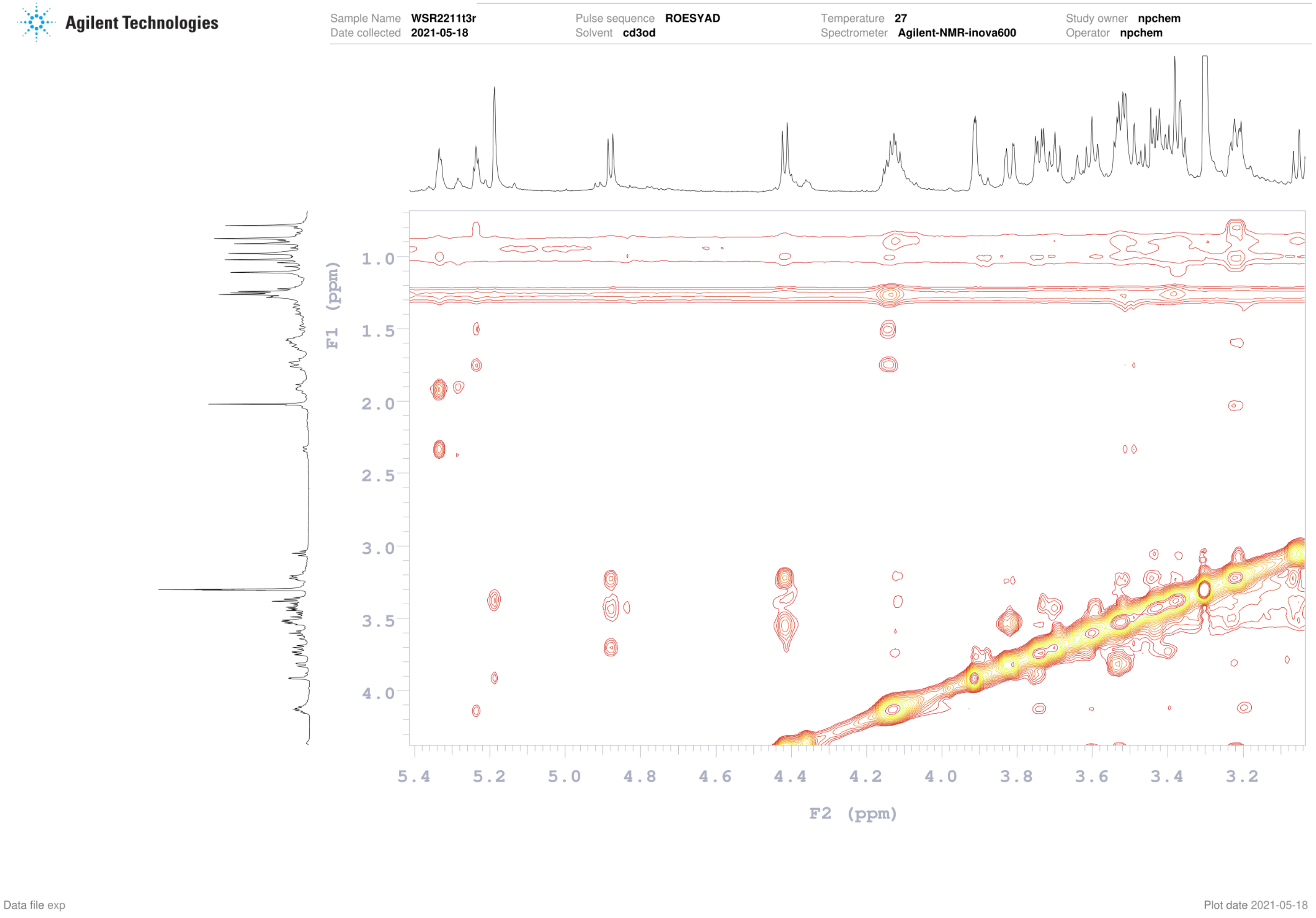

Figure S19 - ROESY spectrum of compound 1 zoomed-in (b)

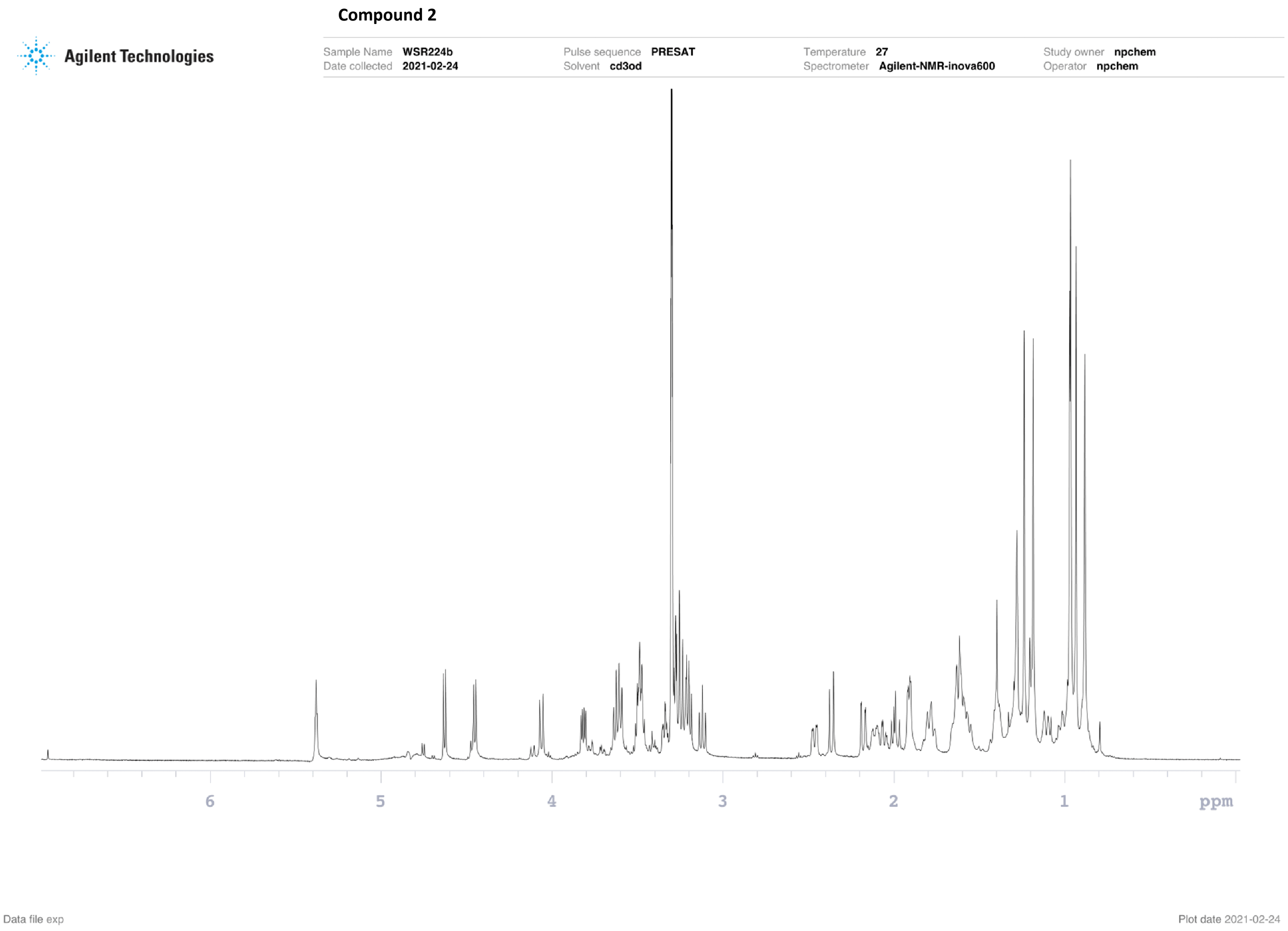

Figure S20 - ^{1}H spectrum of compound 2

Compound 2

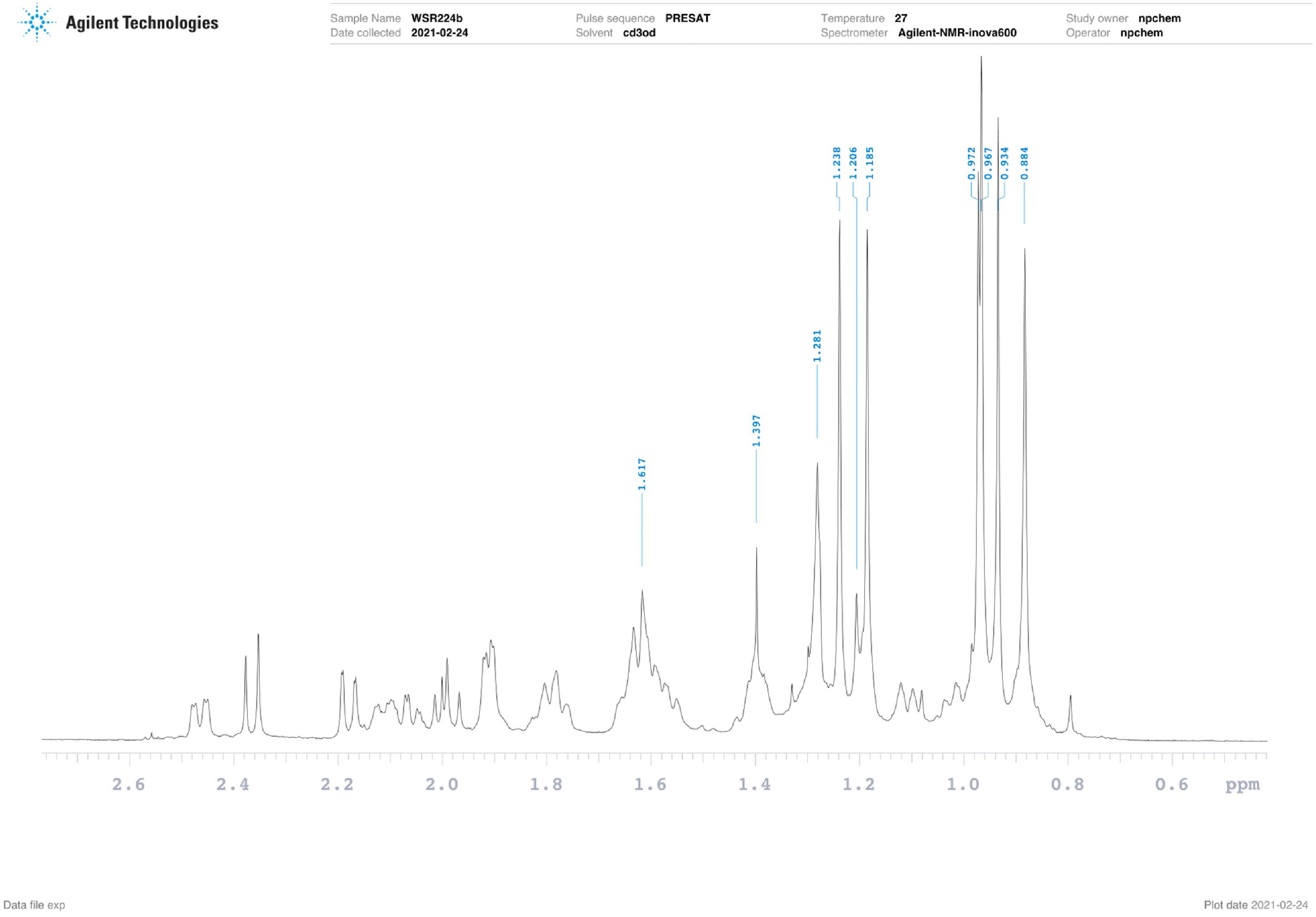

Figure S21 - ^{1}H spectrum of compound 2 zoomed-in (a)

Compound 2

Sample Name	**WSR224b**	Pulse sequence	**PRESAT**	Temperature	**27**	Study owner	**npchem**
Date collected	**2021-02-24**	Solvent	**cd3od**	Spectrometer	**Agilent-NMR-inova600**	Operator	**npchem**

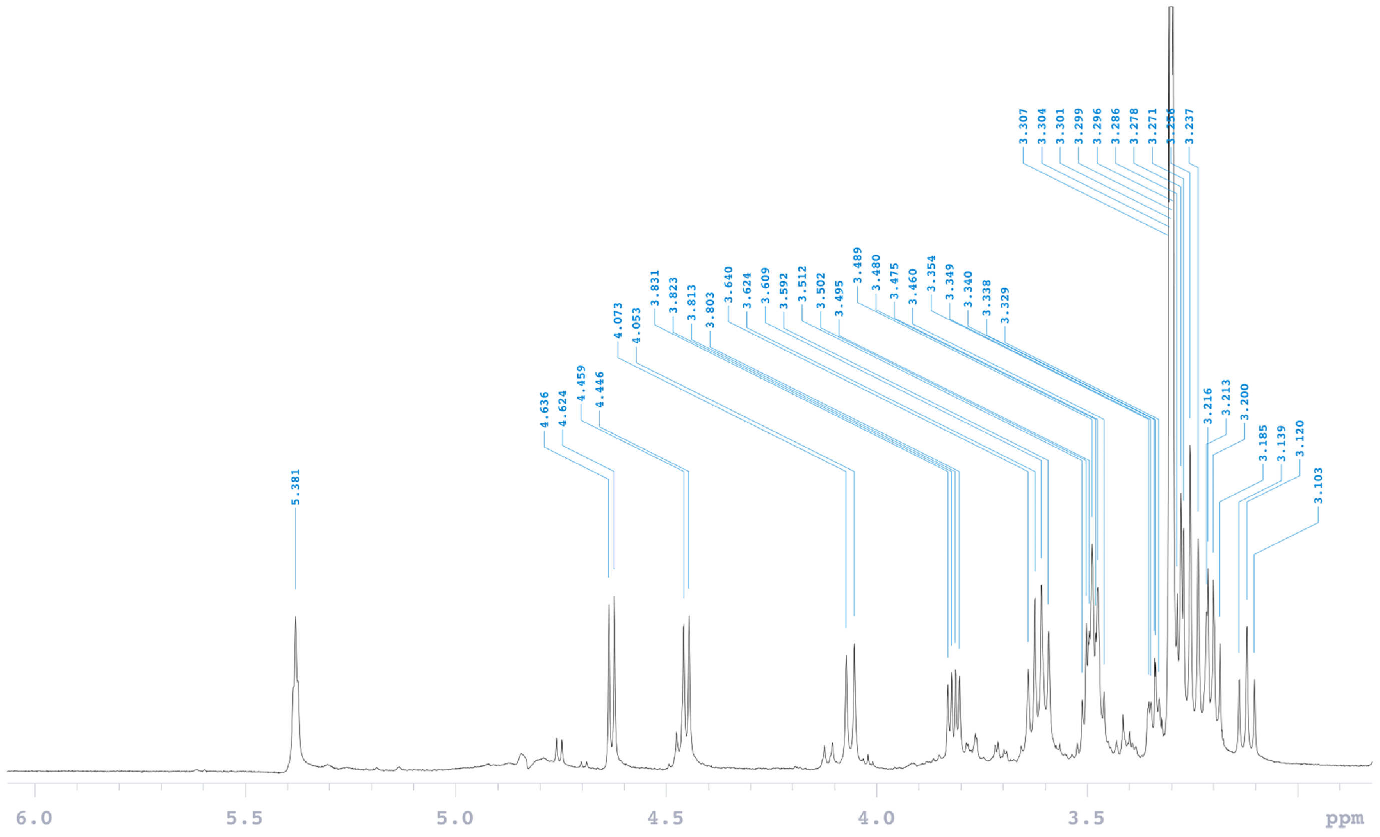

Data file exp

Plot date 2021-02-24

Figure S22 - ^{1}H spectrum of compound 2 zoomed-in (b)

Compound 2

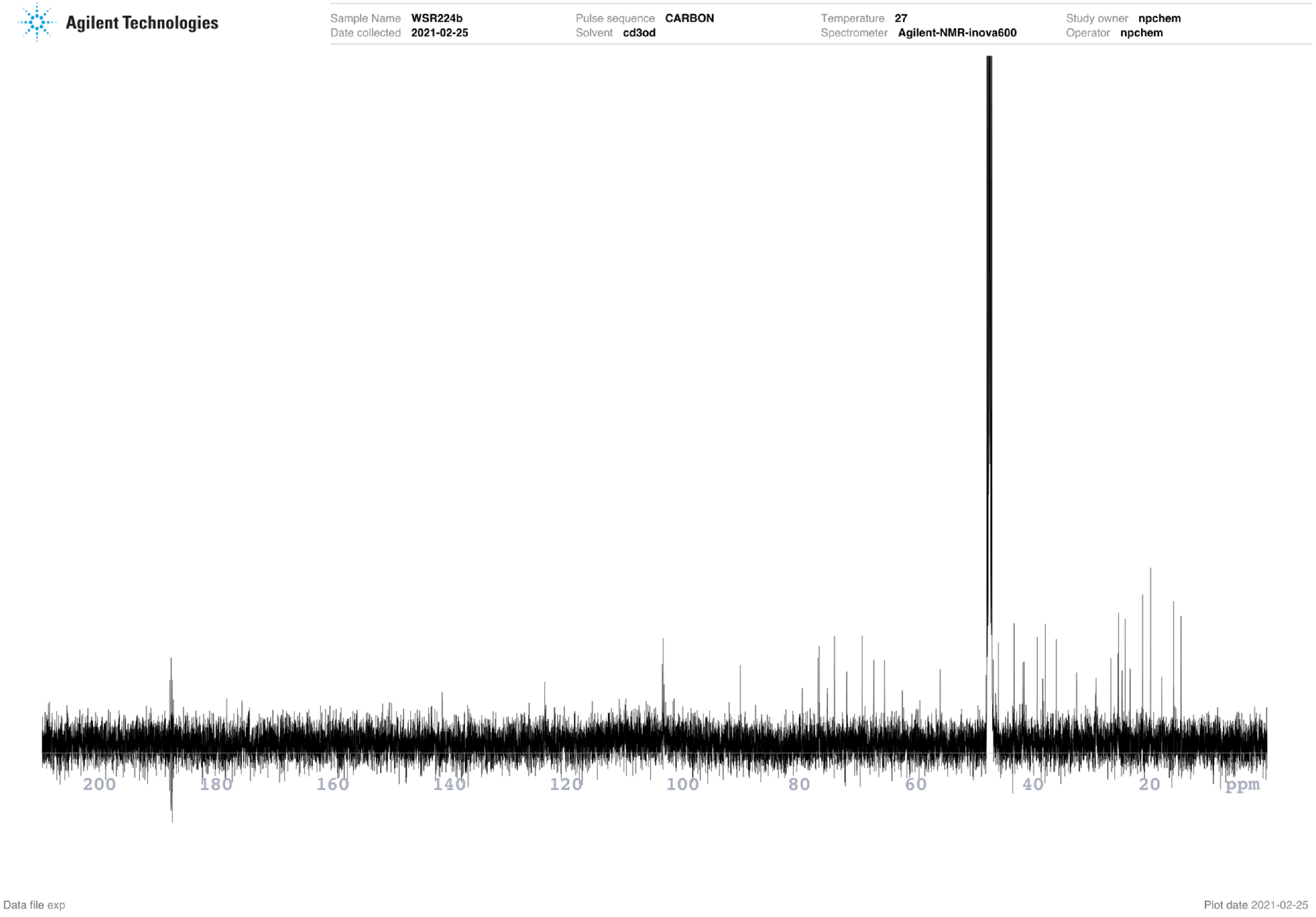

Figure S23 - ^{13}C spectrum of compound 2

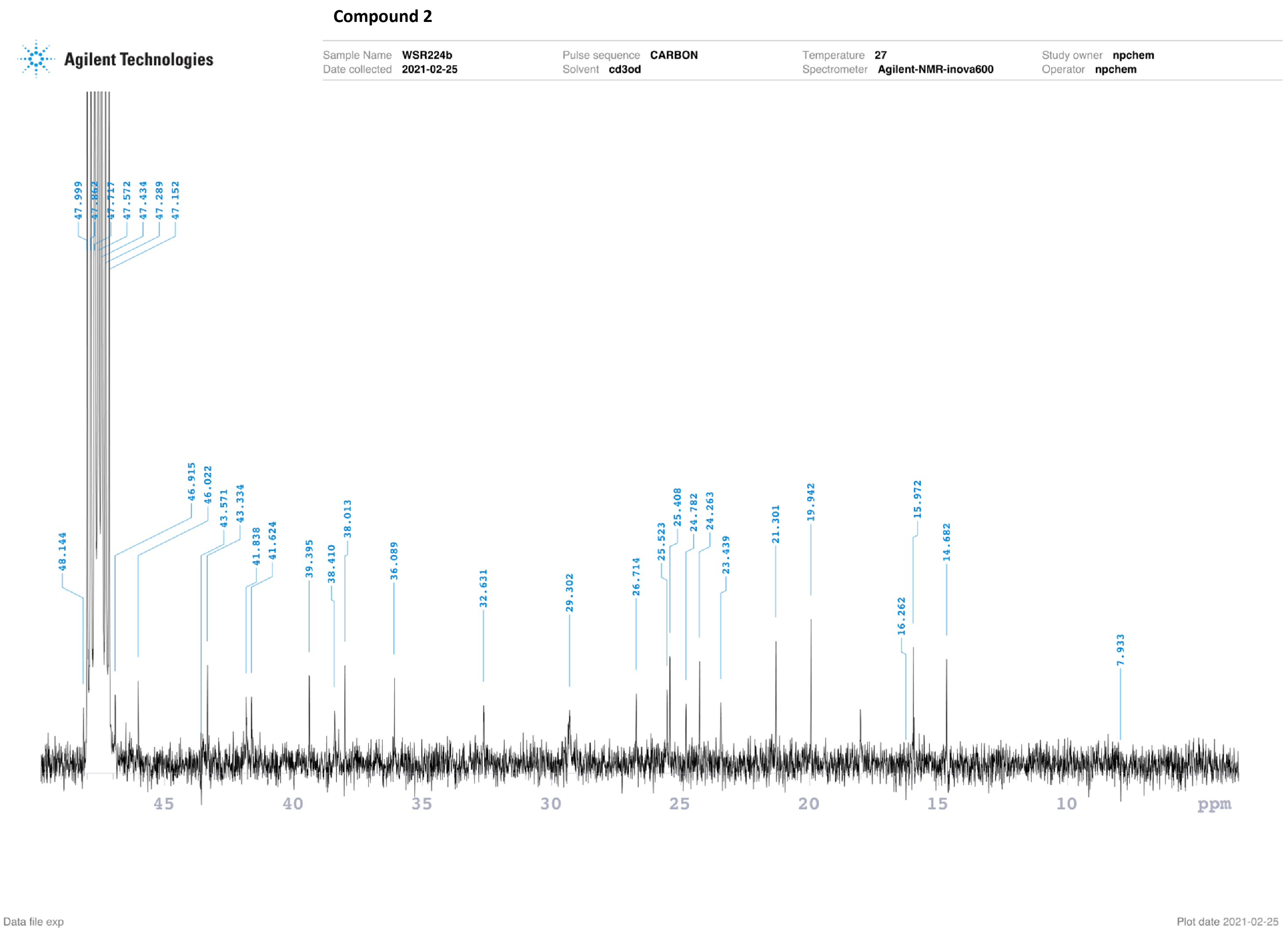

Figure S24 - ^{13}C spectrum of compound 2 zoomed-in (a)

Compound 2

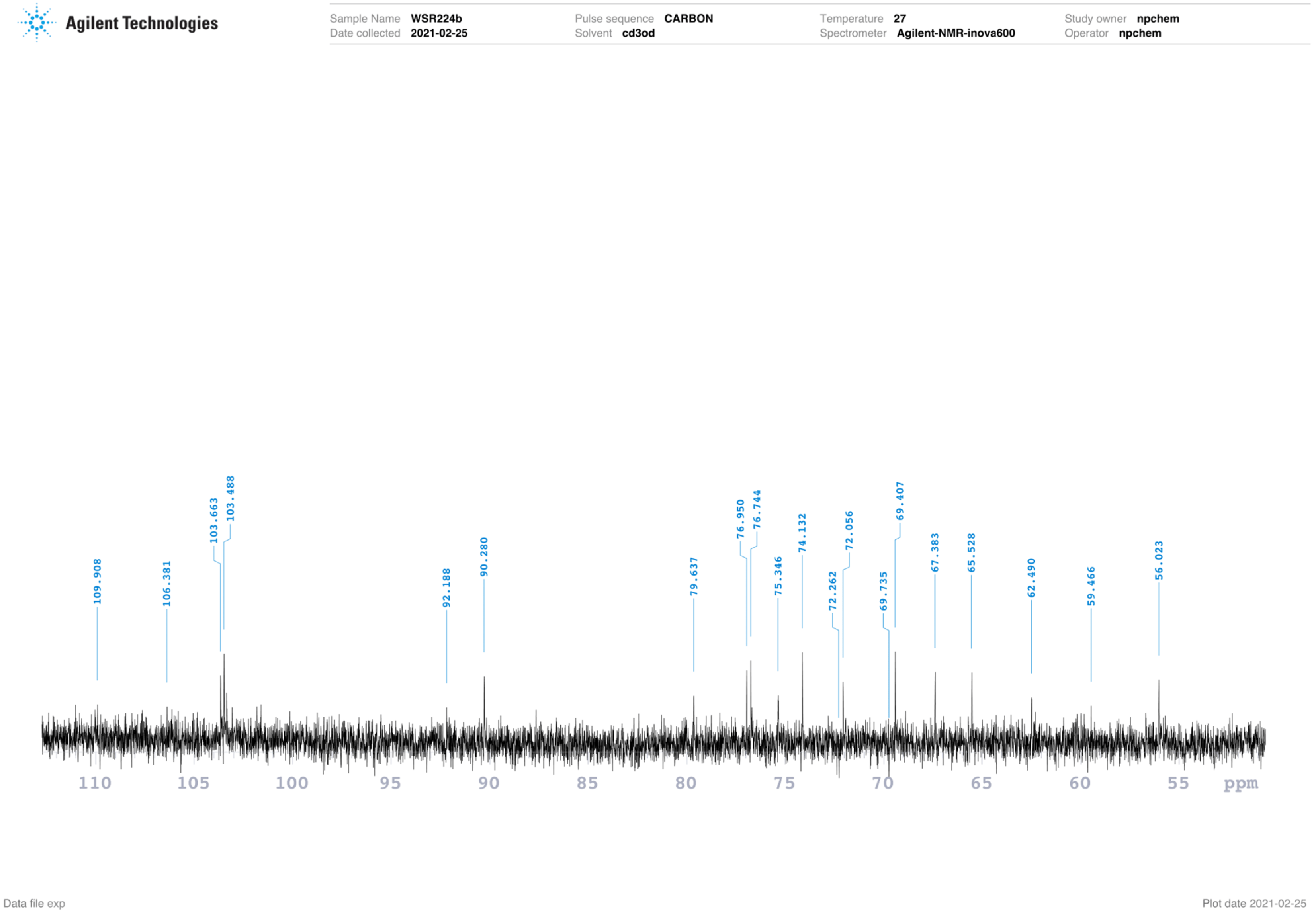

Figure S25 - ^{13}C spectrum of compound 2 zoomed-in (b)

Compound 2

Sample Name **WSR224b**	Pulse sequence **CARBON**	Temperature **27**	Study owner **npchem**
Date collected **2021-02-25**	Solvent **cd3od**	Spectrometer **Agilent-NMR-inova600**	Operator **npchem**

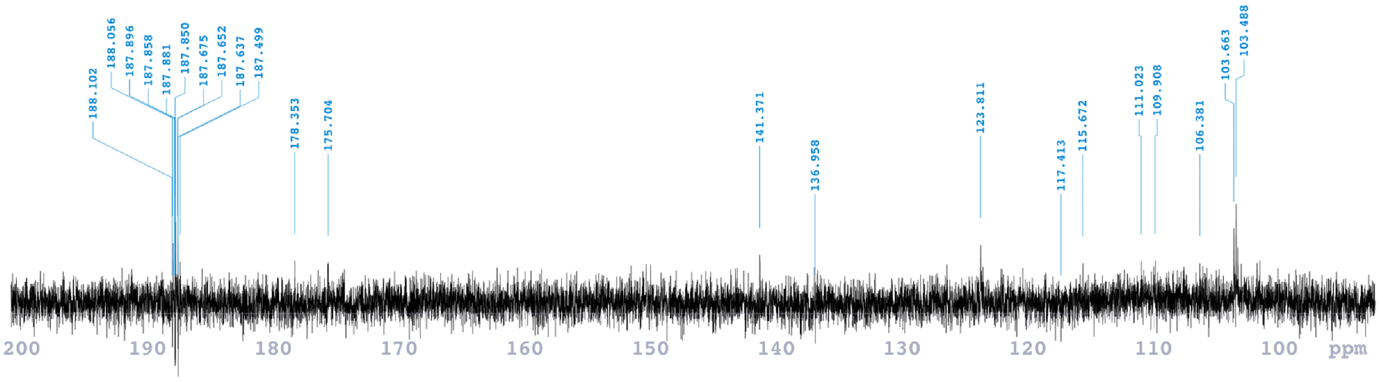

Data file exp

Plot date 2021-02-25

Figure S26 - ^{13}C spectrum of compound 2 zoomed-in (c)

Agilent Technologies

Compound 2

Sample Name **WSR224b**	Pulse sequence **gHSQCAD**	Temperature **27**	Study owner **npchem**
Date collected **2021-02-24**	Solvent **cd3od**	Spectrometer **Agilent-NMR-inova600**	Operator **npchem**

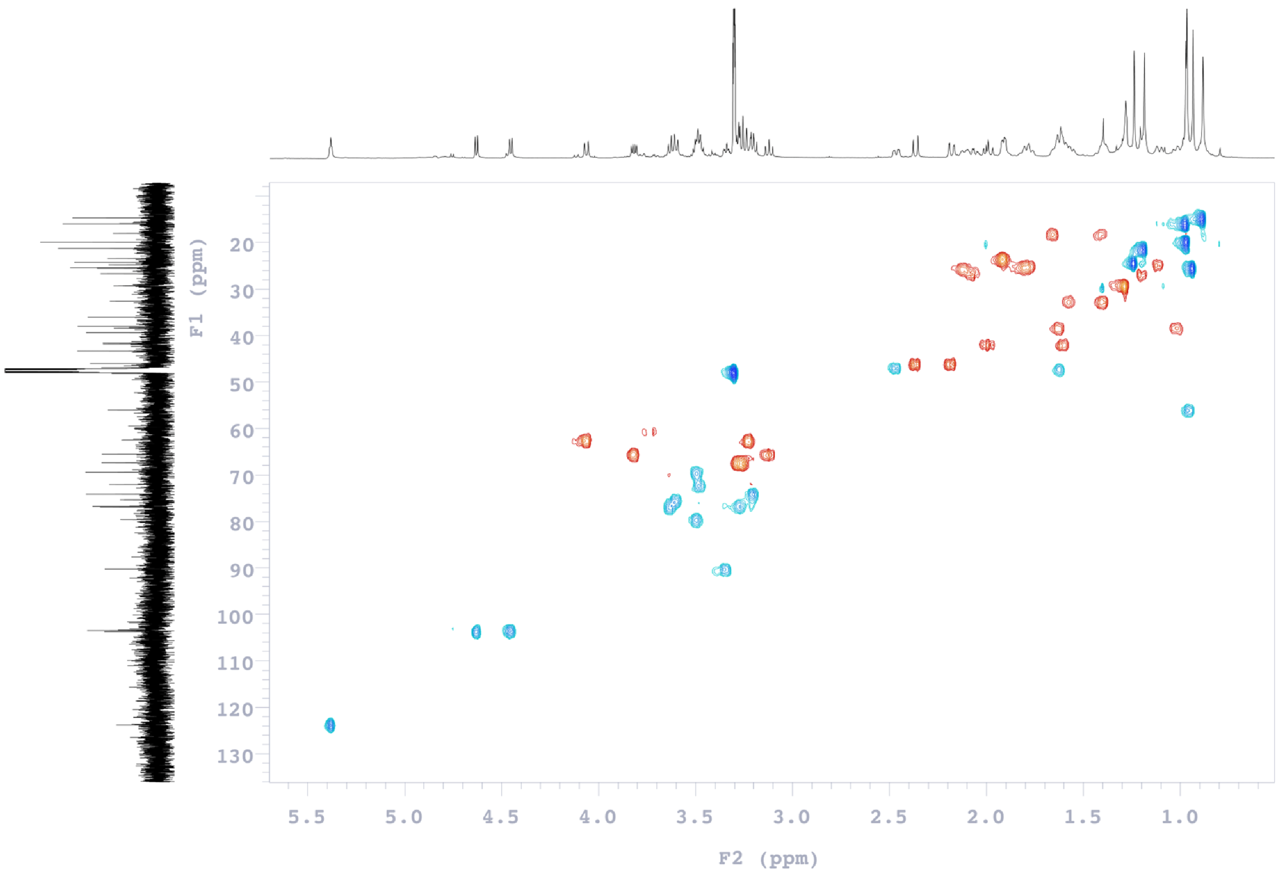

Data file exp

Plot date 2021-02-25

Figure S27 - HSQC spectrum of compound 2

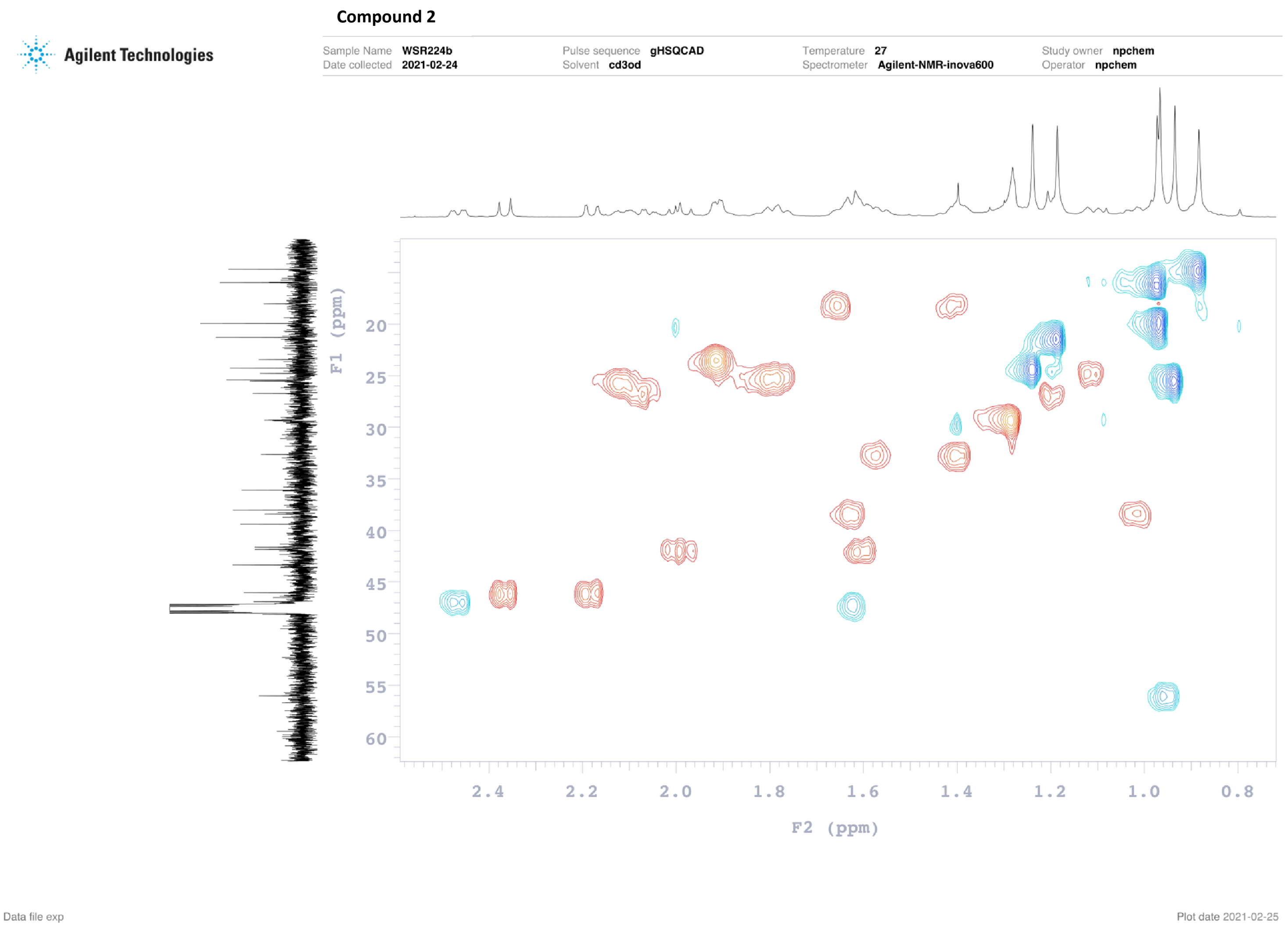

Figure S28 - HSQC spectrum of compound 2 zoomed-in (a)

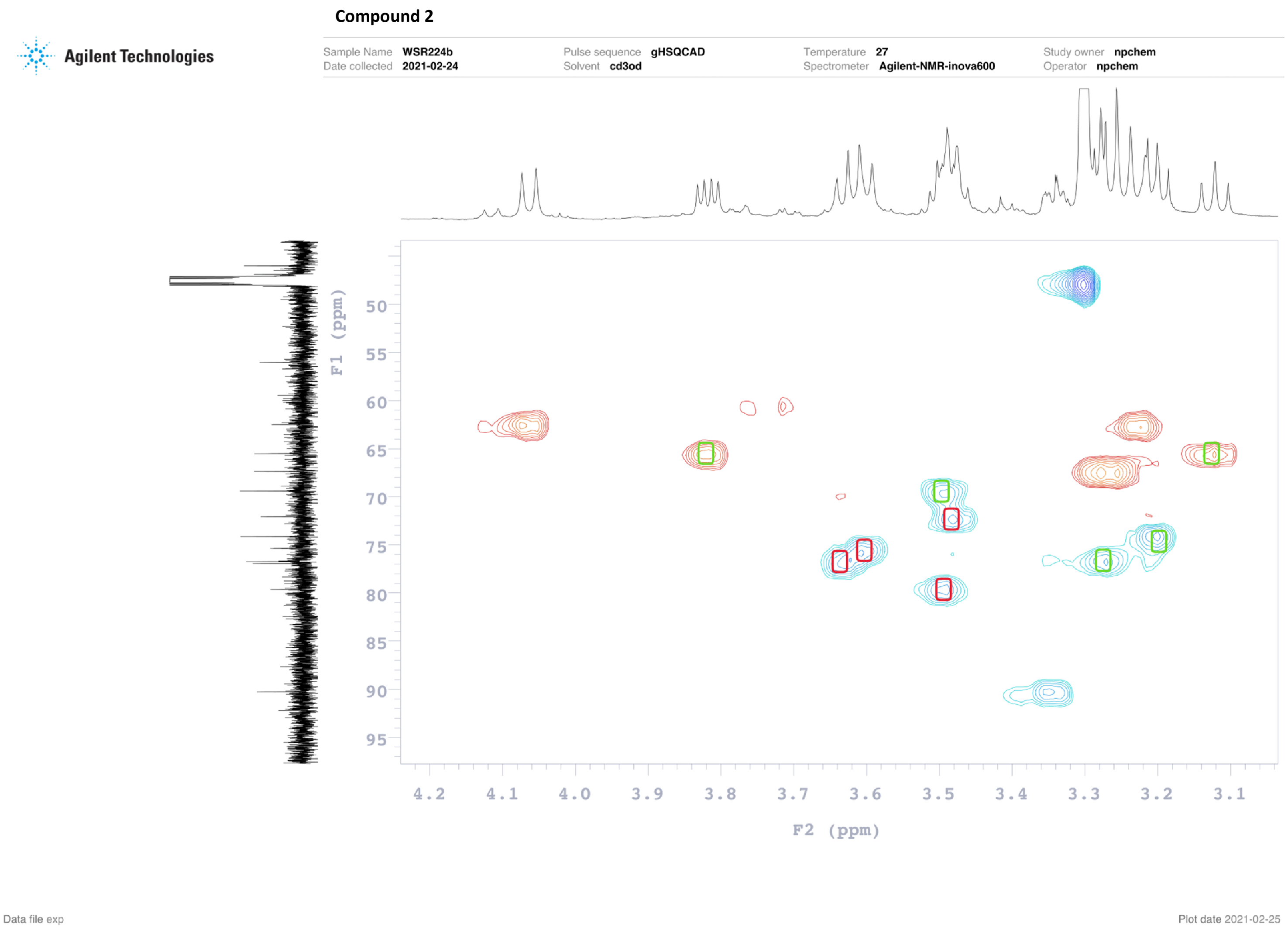

Figure S29- HSQC spectrum of compound 2 zoomed-in (b)

Compound 2

Agilent Technologies

Sample Name WSR224b
Date collected 2021-02-24
Pulse sequence gHSQCAD
Solvent cd3od
Temperature 27
Spectrometer Agilent-NMR-inova600
Study owner npchem
Operator npchem

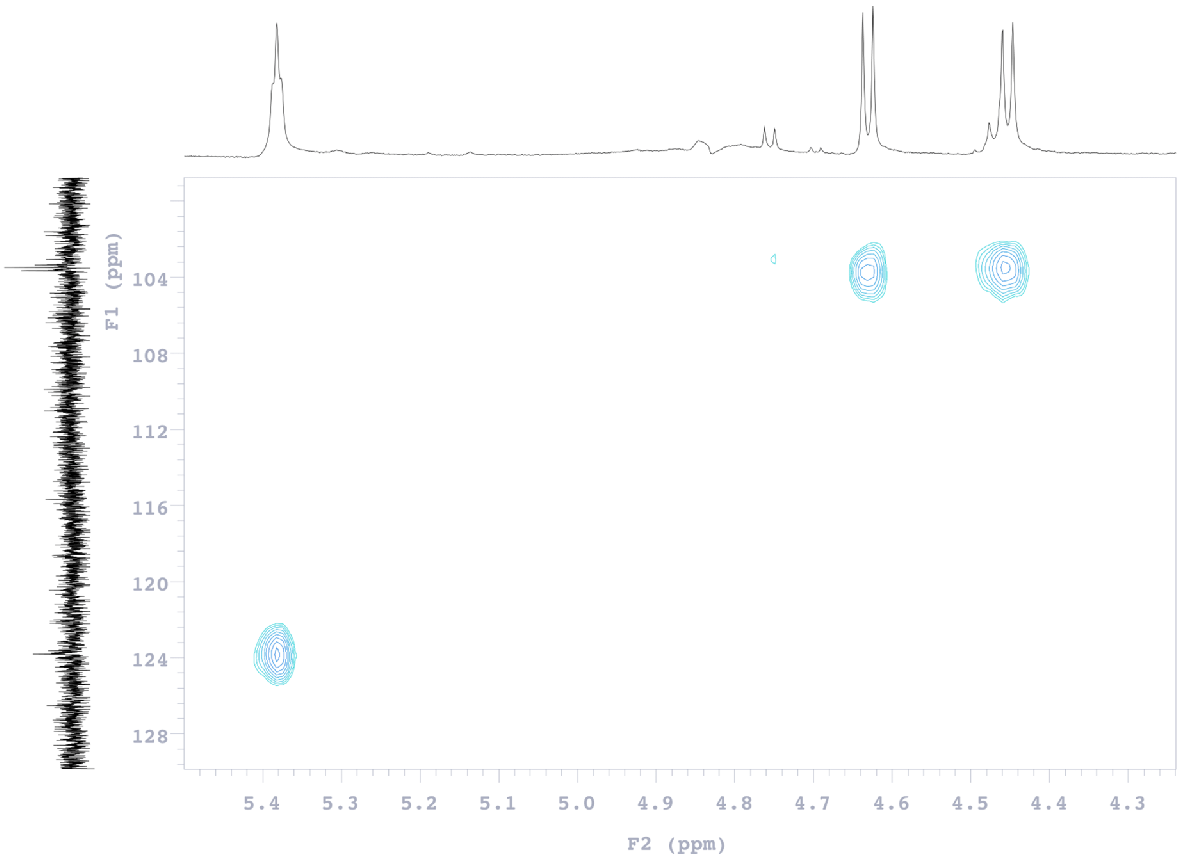

Data file exp

Plot date 2021-02-25

Figure S30 - HSQC spectrum of compound 2 zoomed-in (c)

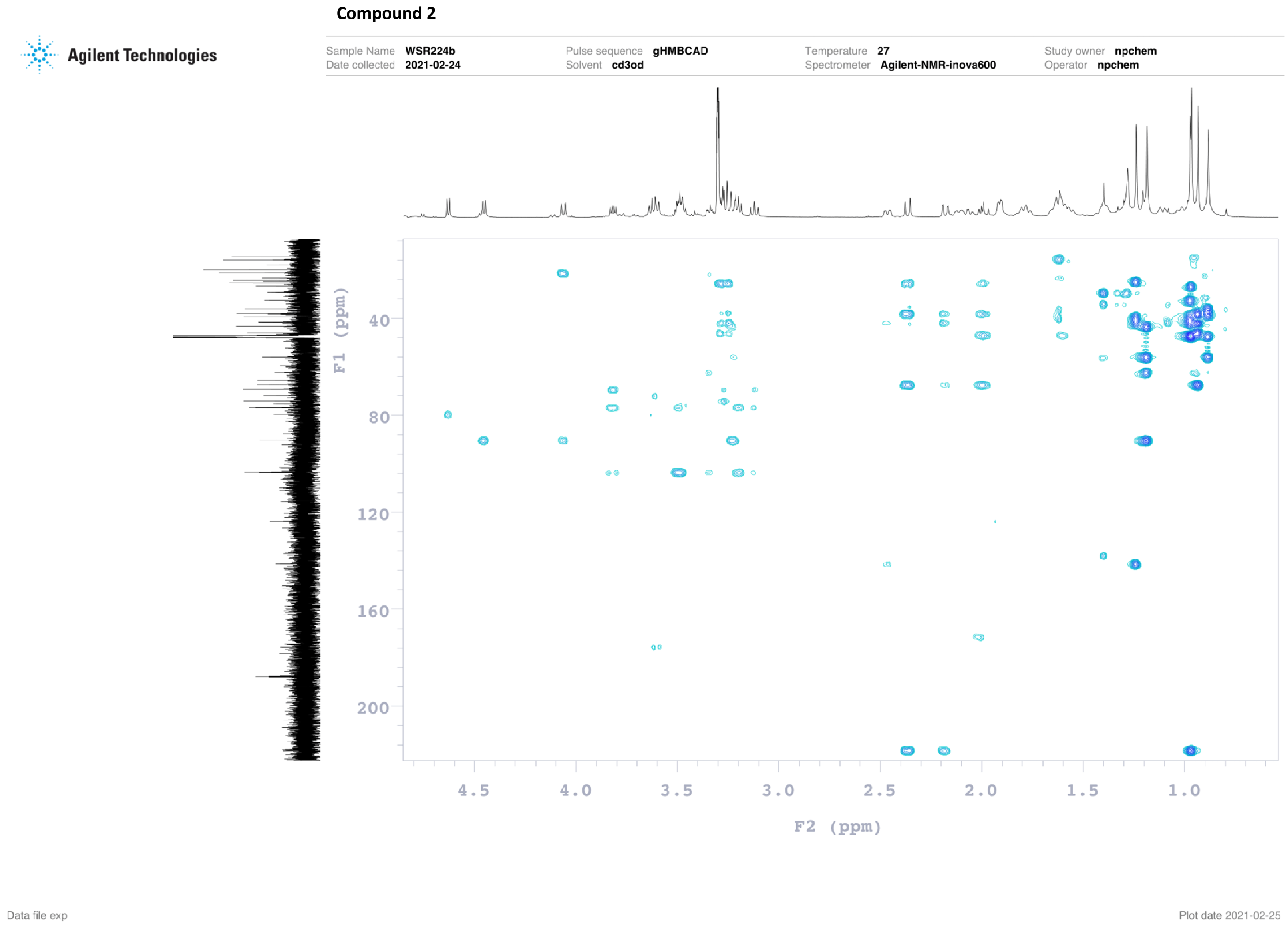

Figure S31 - HMBC spectrum of compound 2

Compound 2

Sample Name WSR224b	Pulse sequence gHMBCAD	Temperature 27	Study owner npchem
Date collected 2021-02-24	Solvent cd3od	Spectrometer Agilent-NMR-inova600	Operator npchem

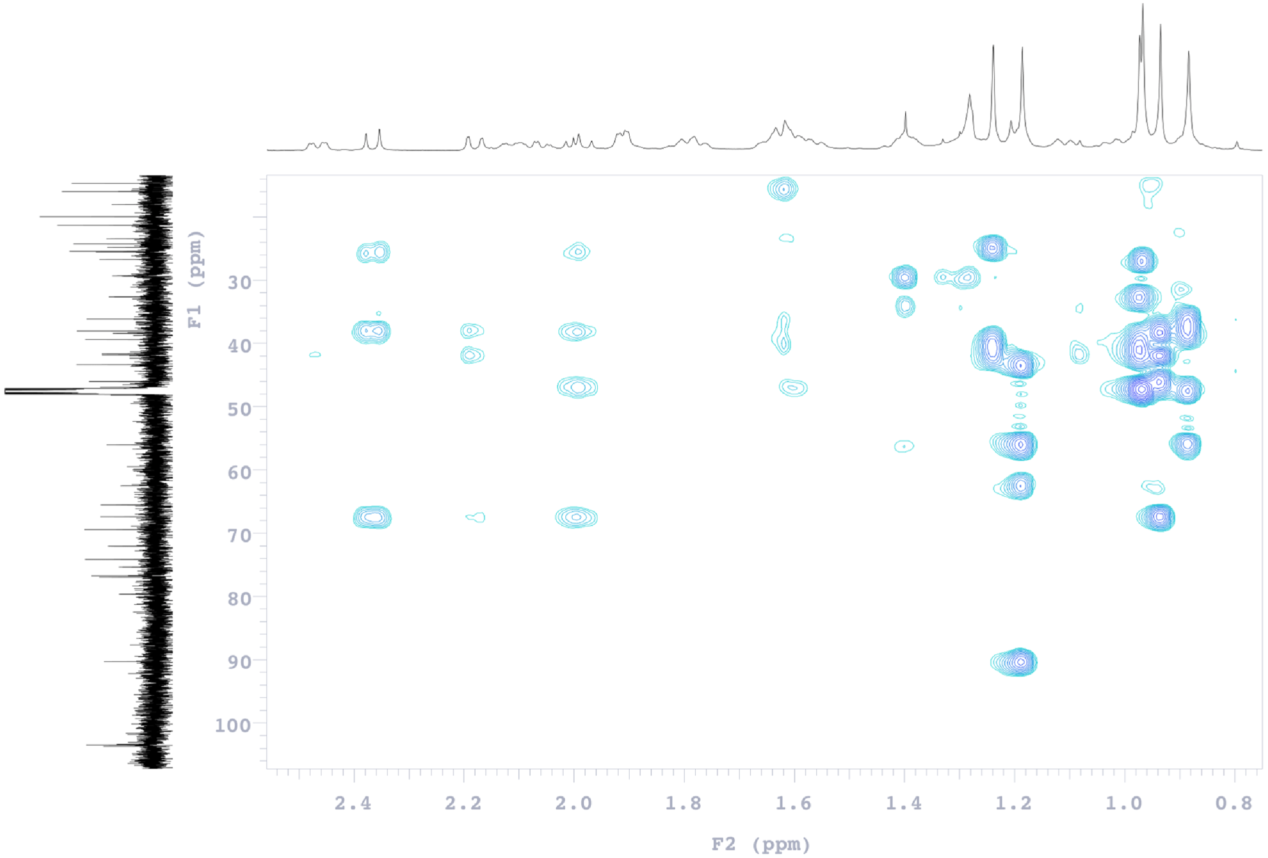

Data file exp

Plot date 2021-02-25

Figure S32 - HMBC spectrum of compound 2 zoomed-in (a)

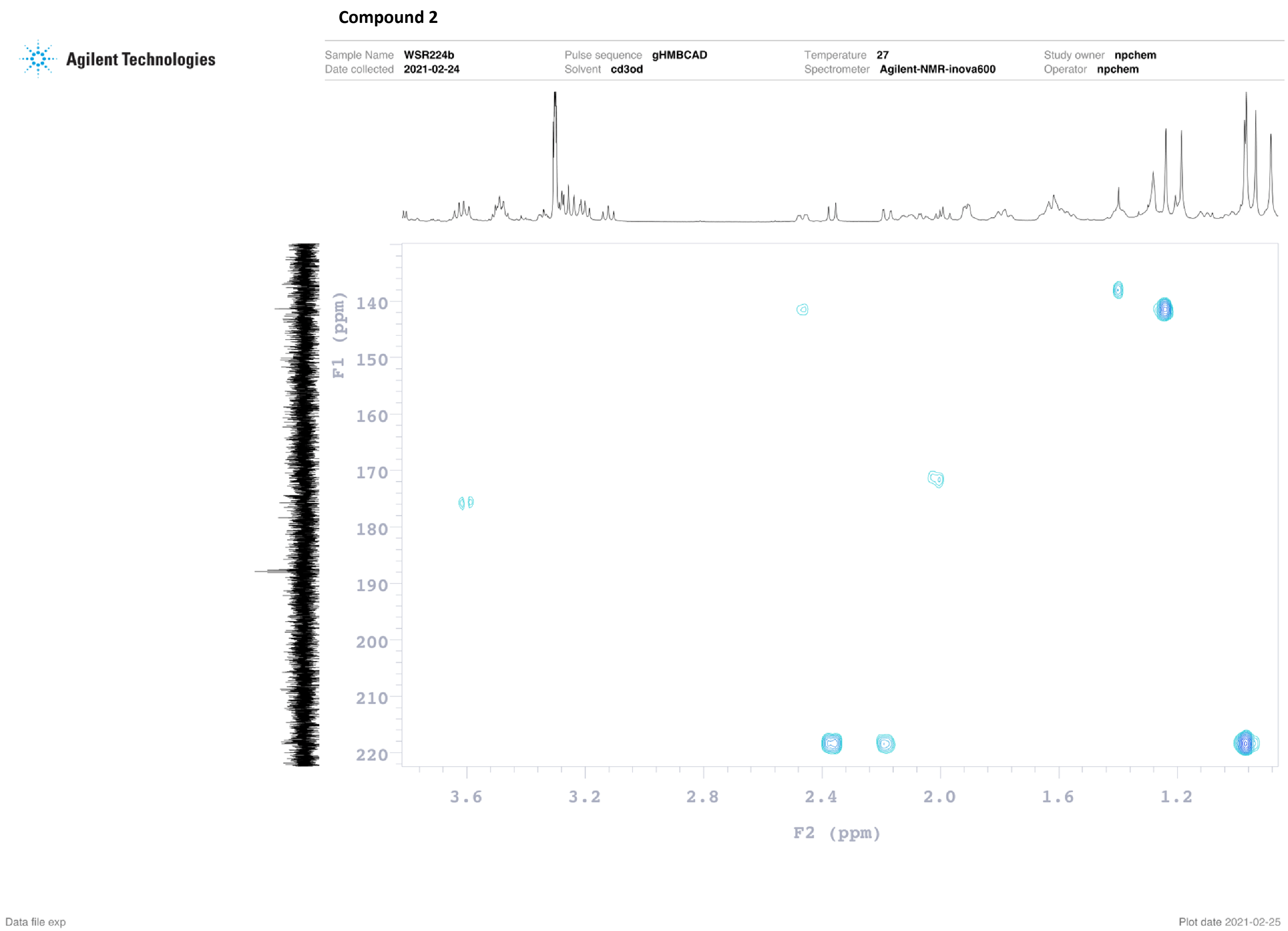

Figure S33 - HMBC spectrum of compound 2 zoomed-in (b)

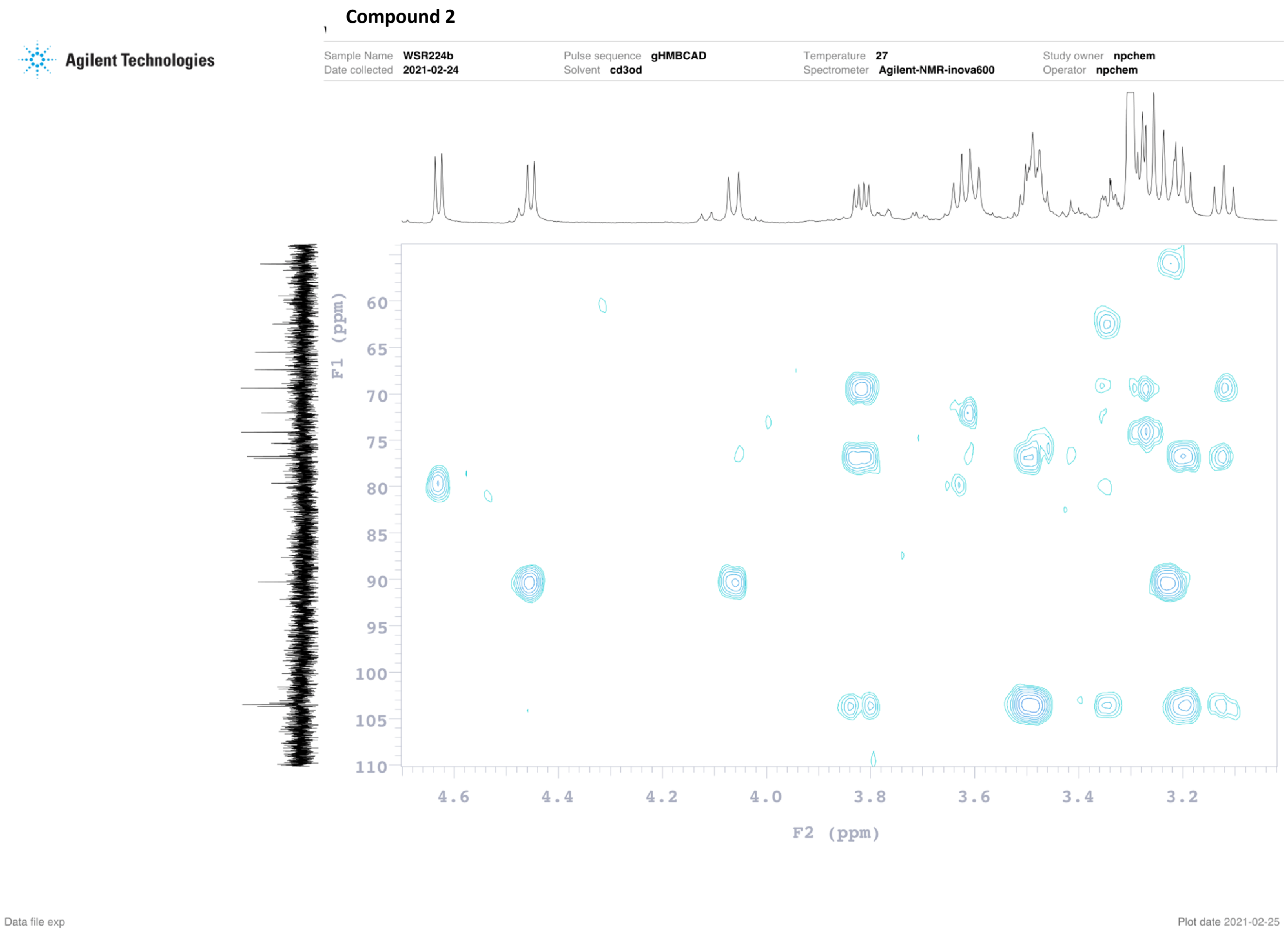

Figure S34 - HMBC spectrum of compound 2 zoomed-in (c)

Compound 2

Sample Name WSR224b
Date collected 2021-02-24
Pulse sequence gCOSY
Solvent cd3od
Temperature 27
Spectrometer Agilent-NMR-inova600
Study owner npchem
Operator npchem

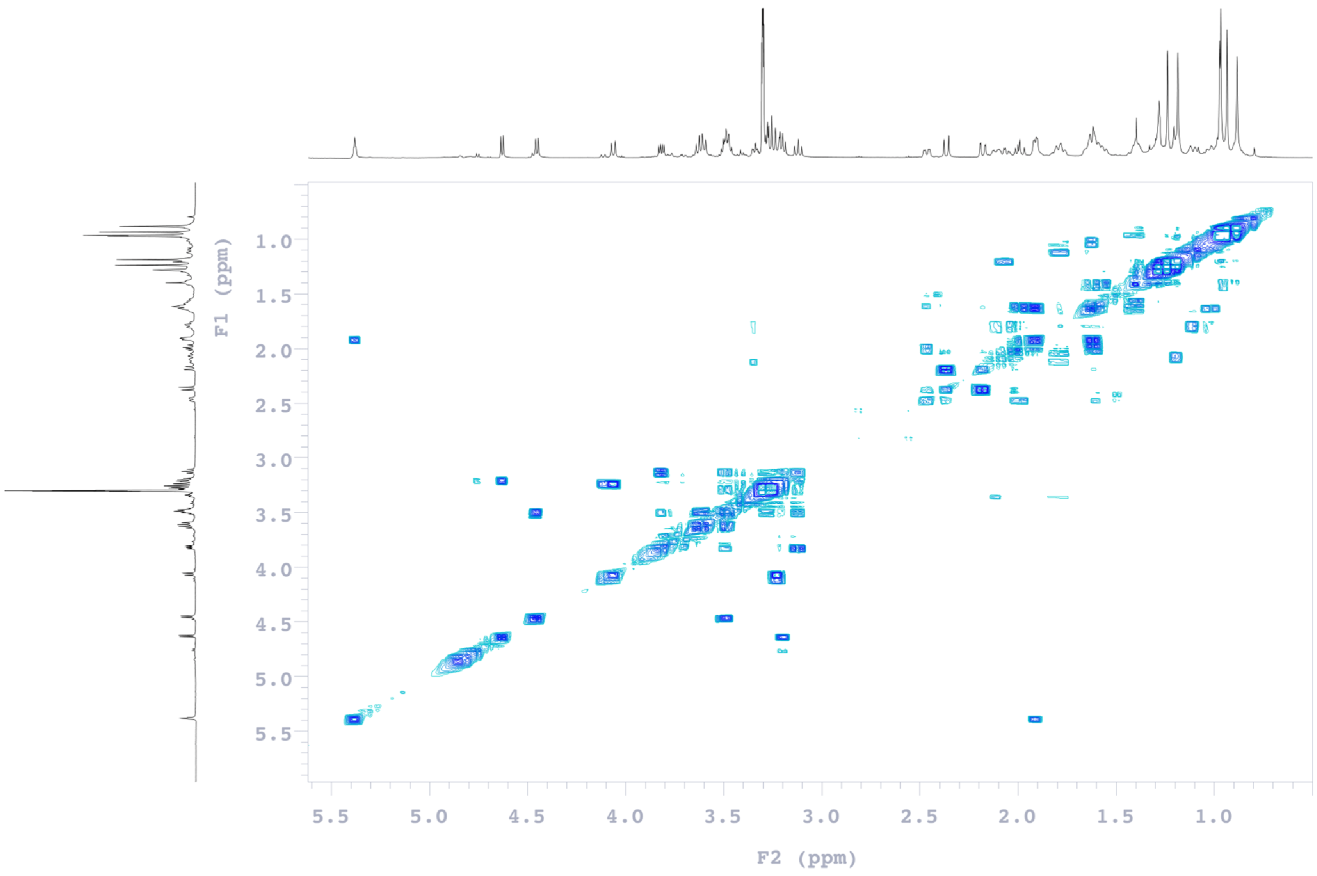

Data file exp

Plot date 2021-02-24

Figure S35 - COSY spectrum of compound 2

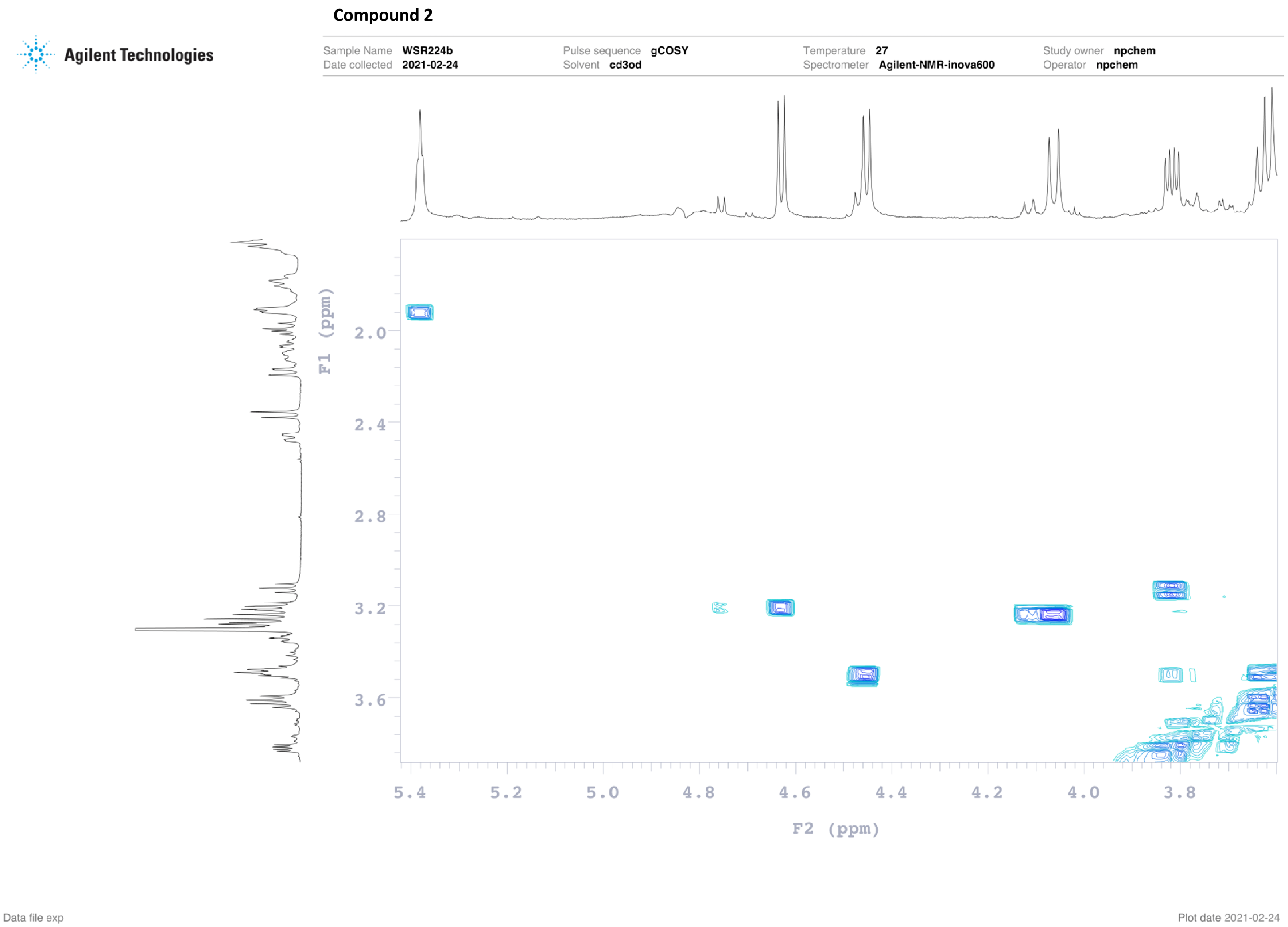

Figure S36 - COSY spectrum of compound 2 zoomed-in

Compound 2

Sample Name WSR224b	Pulse sequence ROESYAD	Temperature 27	Study owner npchem
Date collected 2021-02-24	Solvent cd3od	Spectrometer Agilent-NMR-inova600	Operator npchem

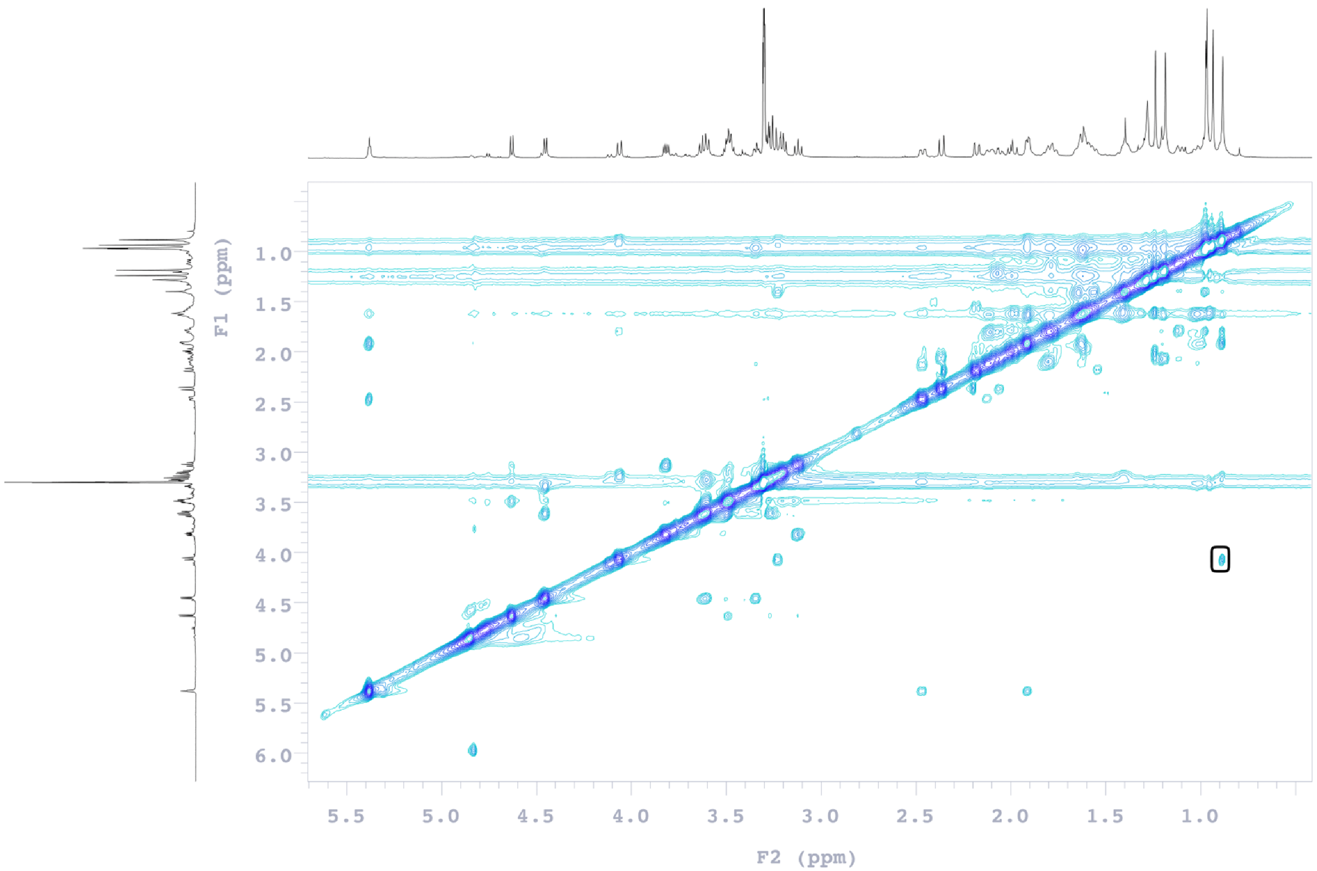

Data file exp

Plot date 2021-02-24

Figure S37 - ROESY spectrum of compound 2

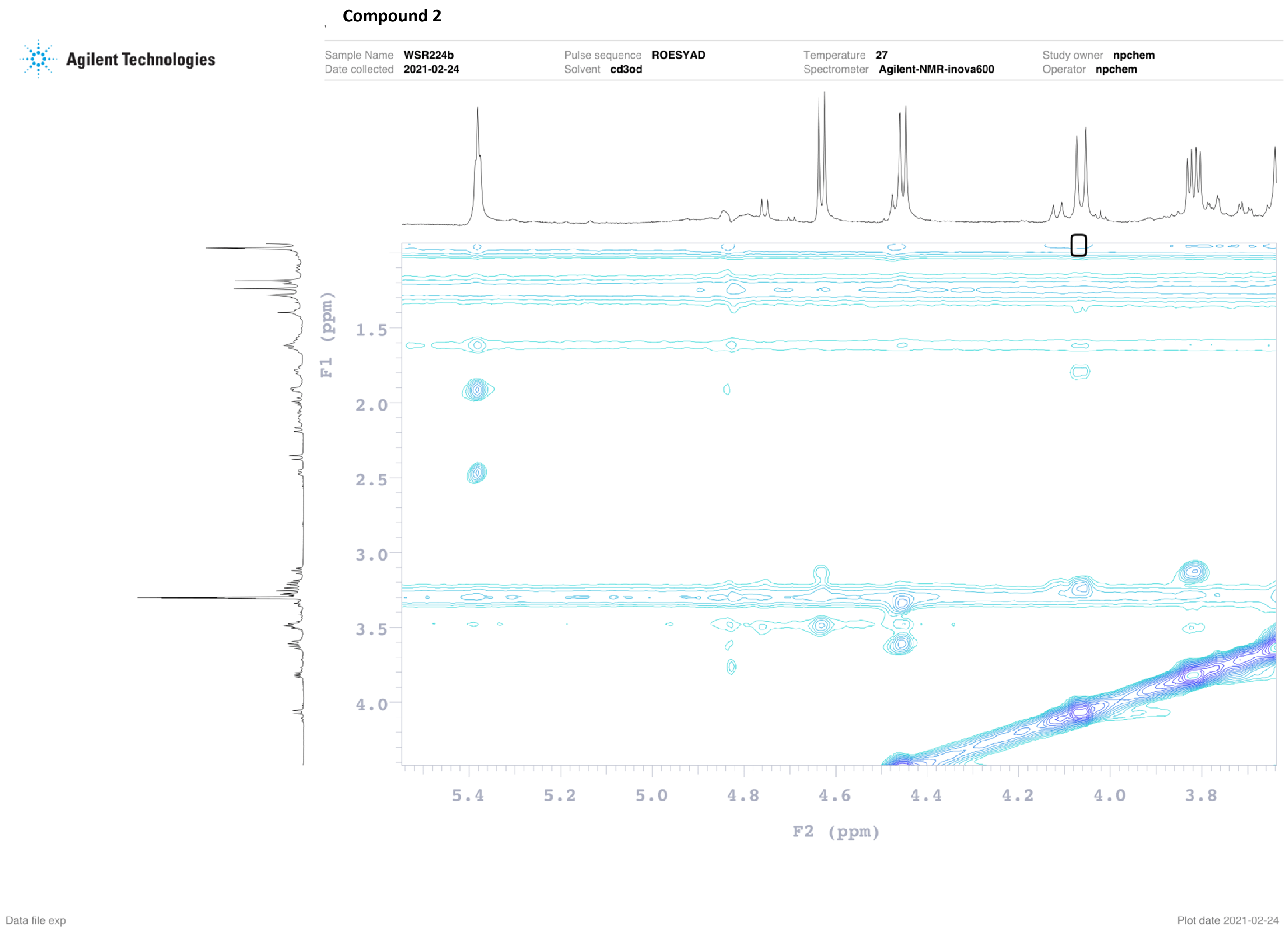

Figure S38 - ROESY spectrum of compound 2 zoomed-in
